# Effectiveness of an mHealth application on remote monitoring and self-management of persons with hypertension in a coastal taluk of Udupi district: A study protocol

**DOI:** 10.12688/f1000research.127131.1

**Published:** 2022-11-28

**Authors:** Prajwal L Salins, Suma Nair, Poornima P Kundapur, Akhilesh K Pandey, Bhageerathy Reshmi, Sabu K Mandapam

**Affiliations:** 1 Health Information Management, Manipal College of Health Professions, Manipal Academy of Higher Education, Manipal, Karnataka, 576104, India; 2 School of Public Health, DY Patil University, Navi Mumbai, Maharashtra, 400706, India; 3 Department of Data Science and Computer Applications, Manipal Institute of Technology, Manipal Academy of Higher Education, Manipal, Karnataka, 576104, India; 4 Department of Community Medicine, Kasturba Medical College, Manipal Academy of Higher Education, Manipal, Karnataka, 576104, India

**Keywords:** mHealth, remote monitoring, self-management, hypertension

## Abstract

**Background:**Hypertension is the most important risk factor for cardiovascular disease, a major cause of death and disability globally. There is increasing evidence that demonstrates clinically relevant benefits from self-monitoring and self-management of blood pressure. Evidence suggests a reduction of systolic BP by 3.2 mm/hg through self-monitoring. The use of mHealth applications in health care monitoring and self-management can help in the timely delivery of health information. Around 33% of Indians use mHealth applications in their daily life. However, well-designed, user complied mHealth applications are essential to reach the masses and to be effective. A previously conducted study in India demonstrated that applications are not customized according to users' needs and expectations and lacked usability assessment by patients. Therefore, we aimed to develop and test a novel mHealth application on remote monitoring and self-management in hypertension.

**Methods**: The study will be carried out in three phases. The first phase will be an in-depth interview to identify the required parameters to develop a customized mHealth android-based application to monitor hypertension. The second phase is to develop the customized application through the Agile development design using the android studio platform. In the third phase, a community-based cluster randomized trial will be carried out to assess the effectiveness of the mHealth intervention on the remote monitoring and self-management of people with hypertension. A sample of 236 people from 12 villages will be randomized and the mHealth application will be delivered to the intervention group and the standard regimen will be continued in the control group.

**Results:**In the proposed study if the intervention is found to be helpful, then hypertension patients in the community can be encouraged to install the mHealth application. This application, if found effective can improve the health status, knowledge, and self-care approach among hypertensive patients.

Registration: CTR India ( CTRI/2022/03/041544).

## Introduction

Hypertension is a major public health concern worldwide and in India with a significantly growing prevalence in both urban and rural communities. It is one of the major causes of mortality and morbidity in elderly people, but hypertension is still a significant health problem ( Karmakar *et al.*, 2018). Approximately 13.5% of global premature deaths are due to increased blood pressure ( Lawes *et al.*, 2008). In low and middle-income nations, the age-adjusted systolic blood pressure (BP) is high and the mean age-adjusted systolic BP has drastically increased in developing countries like East Africa, and South and Southeast Asia. In addition, the number of persons with uncontrolled hypertension rose between 1980 and 2008 due to population growth and aging around the world ( Danaei *et al.*, 2011). Hypertension prevalence in India is enormous. Recent evidence shows a prevalence of hypertension in India of 30.7% ( Ramakrishnan *et al.*, 2019).

Hypertension leads to myocardial infarction, cerebral infarction, heart failure, and kidney disease ( James *et al.*, 2014). Early diagnosis is thus necessary for managing hypertension and preventing future risks ( Dalal *et al.*, 2020). Lifestyle modifications and drug treatment are the two primary types of hypertension care. Managing lifestyle is the primary step in hypertension care for patients over 18 years of age ( James *et al.*, 2014). Lifestyle management including exercise and diet helps in reducing blood pressure ( Lackland & Voeks, 2014). Stress management including various relaxation techniques such as meditation, prayer, and yoga also helps in reducing systolic blood pressure ( Dusek *et al.*, 2008).

There is increasing evidence that demonstrates clinically relevant benefits from self-monitoring and self-management of blood pressure. Evidence suggests a reduction of systolic BP by 2-8 mm/hg ( Chobanian *et al.*, 2003; Tucker *et al.*, 2017). In the recent era, user-friendly technology plays an important role in addressing the challenges with the dissemination of important and relevant health information to the public. mHealth refers to the provision of healthcare services by mobile communications devices ( Santo & Redfern, 2019). The increase in the use of smartphones and tablets has been accompanied by an increase in the use of mHealth applications. The use of mHealth in the self-care of chronic diseases such as hypertension is becoming increasingly popular. Most mHealth apps are designed to assist persons with hypertension in self-management by providing self-monitoring tasks, alerts, personalized information, and feedback ( Santo & Redfern, 2019).

In India, the awareness, medical treatments, and control of hypertension remain poor despite its substantial prevalence. Around 33% of Indians use some mHealth application in their daily life. Although few applications are available in the market, there are no adequate applications customized to the Indian population ( Santo & Redfern, 2019). A recent study conducted demonstrated that applications are not customized according to Indian users’ needs and expectations in regional language and lacked usability assessment by patients ( Alessa *et al.*, 2018; Santo & Redfern, 2019). Most of the evidence that determines the effectiveness of mHealth applications has been conducted in developed countries with very little evidence from developing countries, specifically the South Asian region and India in particular.

Therefore, there is a need to develop an application that meets the local health information needs, provides information in the local language, and is designed based on patient usability feedback. Further studying the effectiveness of the application on knowledge about the disease, self-care among the participants, and blood pressure status of the patients with hypertension will throw more light with regards to this.

Thus, we aim to develop a user-friendly mHealth application for remote monitoring and self-management in persons with hypertension and assess its effectiveness. Further, we propose to test this application for its effectiveness in improving the knowledge, self-efficacy, health status, and self-management of persons with hypertension.

## Methods

### Ethics and registration

The approval has been obtained from Institutional Ethics Committee (IEC No: 587-2021), and written consent from the participants will also be obtained. The study has been registered in Clinical Trial Registry - India ( CTRI/2022/03/041544).

### Phase 1

The study is proposed to be conducted in three phases. The first phase of this study will be a qualitative one, to identify the needs and expectations of people with hypertension in the Udupi district, Southern India. This phase will be carried out in three steps:

1. Focus group discussions (FGDs) with persons diagnosed with primary hypertension
2. In-depth interviews with general physicians and cardiologists
3. Development of educational material

*Focus group discussions*

For the FGDs, we will be including persons diagnosed with primary hypertension and who are being medically managed (ICD 10 code: I10), between the age of 18 and 60, of either gender, and have access to smartphones (Android) with internet connectivity. The exclusion criteria are people with visual impairment, who cannot read and comprehend either English or Kannada, and who are dependent on self-care. We will also be including caregivers of hypertensive patients above 18 years of age, who have had experience for more than a year and can understand English or Kannada, and have access to Android-based smartphones with internet connectivity. For this FGD, we will have a sample size of 8 participants. This is as per previous studies.

**Figure 1.**
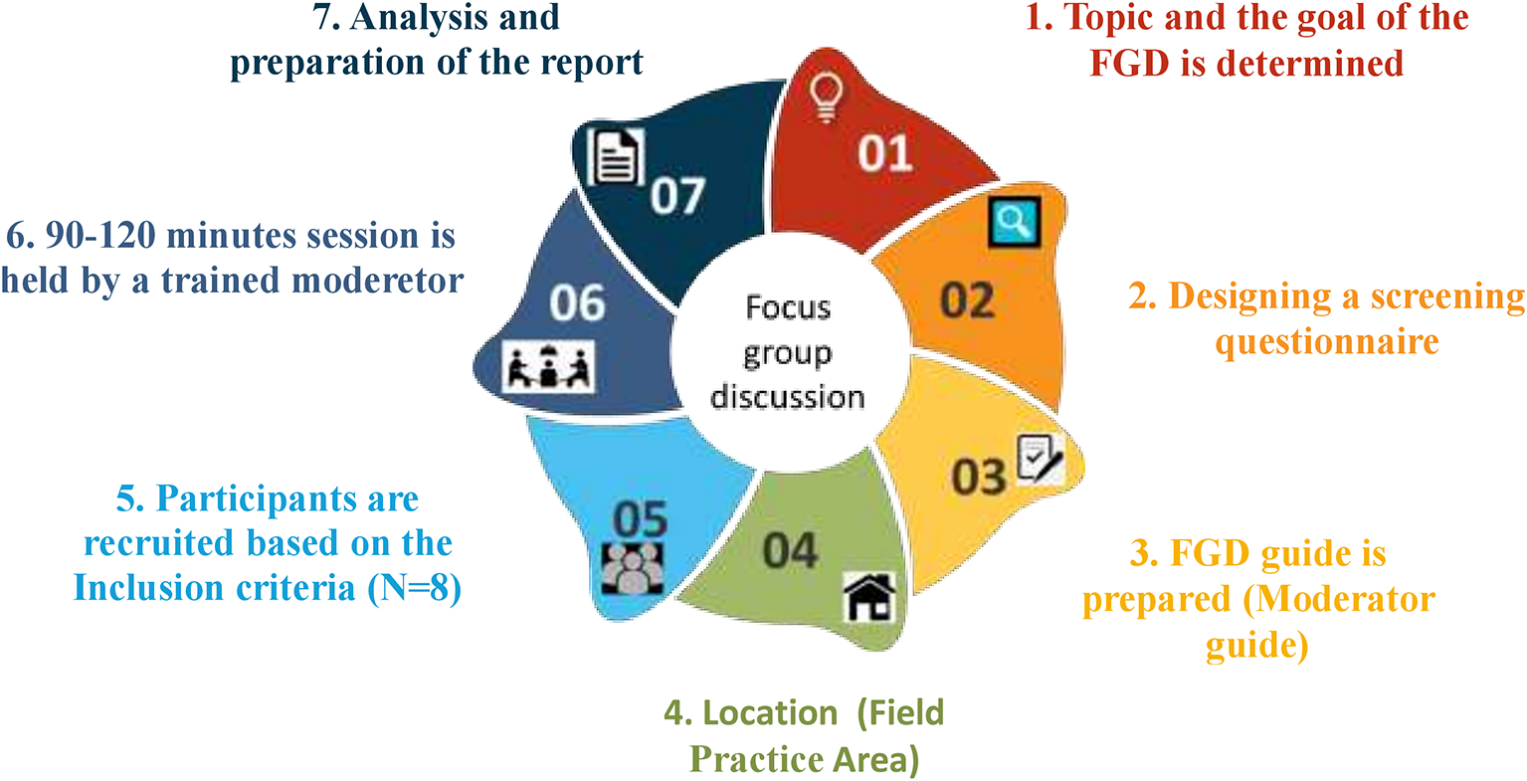
The process of focus group discussions.

The participants will be screened and recruited from health centers of Udupi district, Southern India. They will be contacted in person by the primary investigator and the written informed consent will be obtained from eligible participants after explaining the study. The recruited participants will be asked to take part in FGDs which will be carried out for the proposed objective. The moderator, who is the primary investigator, and the assistant will be introduced to the participants and the purpose of the FGD, as well as the guidelines, will be briefed to them. Before the FGDs, the screening questionnaire will be designed based on the objective of the study and validated by five experts in the field of community medicine and qualitative study experts. Subsequently, a moderator guide will be designed which includes the research rules, explanation of confidentiality, introductions to the FGD, questions, probes and follow-up questions, and conclusion. The FGDs will be audio recorded and conducted in the community health center till thematic saturation is achieved. We will be conducting thematic analysis using ATLAS.ti software version 9 and the report will be prepared. The process of the FGD has been depicted in Figure 1.

*In-depth interviews*

**Figure 2.**
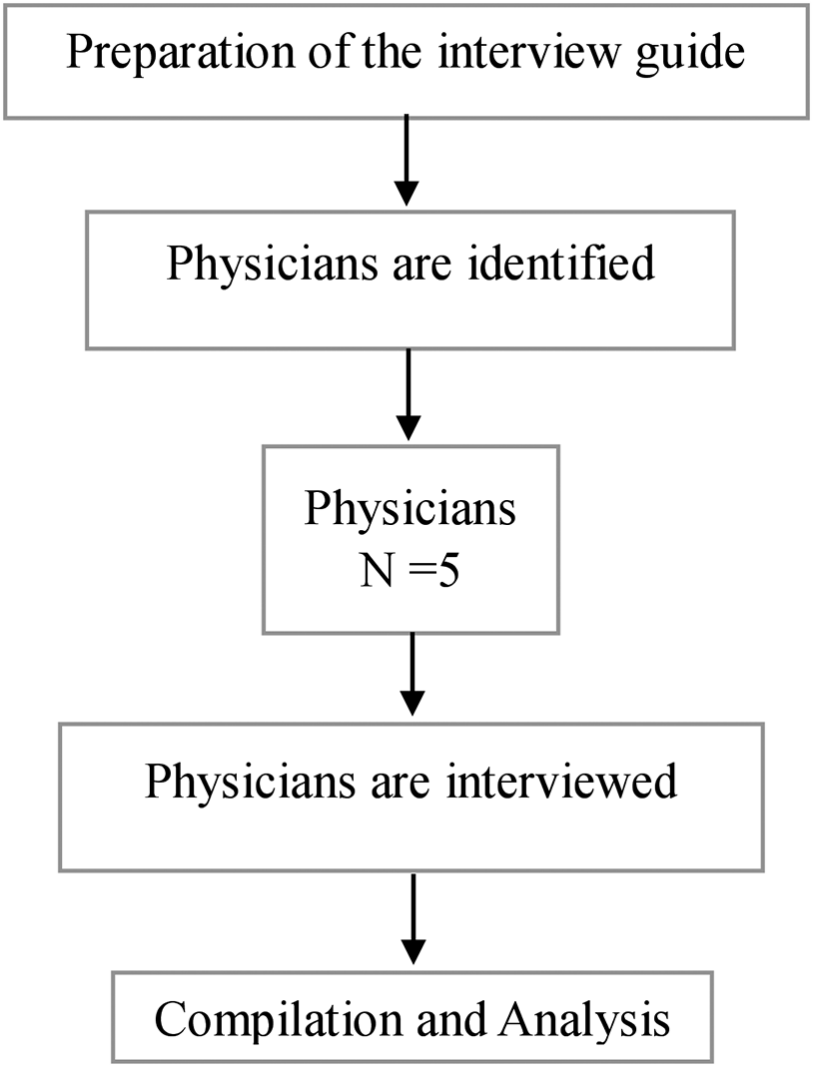
The process of in-depth interviews.

An in-depth interview guide will be prepared separately for physicians and an interview will be conducted to know their views, ideas, and opinions on the proposed mHealth application. A total of five physicians will be approached in person for the in-depth interview. Interviews will be conducted in the OPD by the primary investigator. The interview will be audio recorded. Thematic analysis will be carried out using ATLAS Ti version 9. The obtained information will be compiled and will be incorporated into the mHealth application. The process of in-depth interview is shown in Figure 2.

*Development of educational information module on self-management for persons with hypertension*

Educational information material will be prepared based on the literature review and according to recommendations for the conception and efficacy of educational tools referring to content, language, organization, layout, illustration, learning, and motivation. The developed educational information material will be translated and back-translated to and from Kannada. The information will be content reviewed using PEMAT-A/V by five physicians and five health information professionals and any comments will be considered while preparing the final version ( Shoemaker *et al.*, 2014).

### Phase 2

This phase of the study will be to develop a mHealth application for self and remote monitoring and self-management of hypertension and pilot test the application (to assess the acceptability, feasibility, usability, and user-friendliness of the app). Agile development design will be used to develop the application using Android studio ( Moe *et al.*, 2010).

The mHealth application will be developed in five steps as follows:

*Requirements analysis*

Information based on end users' needs and expectations about the mHealth application will be captured as per the requirements to develop the mHealth application (data will be captured in phase 1 of the study).

*Designing the requirements*

Team members (i.e. Health Information Professionals, Physicians, Cardiologists, and Software developers) will be identified and the requirements of the end users will be discussed. Based on the requirements, high and low-level designing of the mHealth application will be done.

*Development*

The mHealth application will be developed based on the requirements specified by the end users. This step includes coding the application based on the design. Android Studio, an open source for Android software development will be used to develop the proposed mHealth application. The mHealth mobile application will connect to a backend database application (web-based) to help administration of content, visualization, and analysis of the data collected.

*Testing and quality assurance*

The developed application will undergo standard testing and a quality assurance process to identify and rectify the issues and bugs.

*Pilot testing and feedback*

A pilot test will be carried out once the application is developed and will be installed in Android-based smartphones among persons with hypertension to assess the acceptability, feasibility, usability, and user-friendliness of the application. Feedback from the end-users about the mHealth application would be obtained for necessary modifications in the application.

*Deployment*

The developed application will be ready for use by persons with hypertension after pilot testing for acceptance and use for self-monitoring and self-management of hypertension.

**Figure 3.**
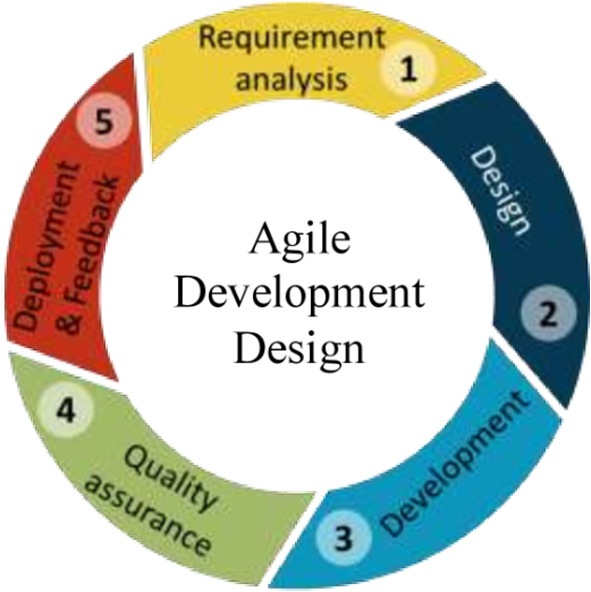
The process of Android software development.

The steps for the development of the software is shown in Figure 3.

Figure 3 shows the process of android software development.

Finally, requirement analysis will be carried out by the investigator along with experts by obtaining the requirements from the end-users. Based on the requirement, an investigator will design the mHealth application which includes various modules, layouts, tabs, widgets, and contents. The coding of the application will be done by the software developer with the help of an investigator. The developed application will be tested for quality assurance and will be pilot tested among the participants. The investigator will be involved in the front and backend process of software development.

The application will consist of blood pressure monitoring (Bluetooth enabled as well manually entered), weight, height, body mass index (BMI), medication reminder and physical activity (step count), and graphical reports of BP and weight. Weekly and monthly summaries (which can be converted into PDF and shared), abnormal warnings, Dietary Approaches to Stop Hypertension (DASH) diet plan, health education material, and recent updates on hypertension management will also be provided.

### Phase 3

An open-label **,** parallel cluster randomized trial will be conducted from October 2023 to March 2025 with villages as the unit of randomization into the intervention and control arm with a 1:1 allocation. 12 villages in the Udupi district will be considered as the clusters included in the sampling frame and eligible for randomization. People diagnosed with hypertension within these clusters will be the targeted population. The eligibility criteria for the clusters include a hypertensive population strength ≥ 10. In case a cluster fails to have the prescribed strength, it will be clubbed with an adjacent cluster to achieve the required number. For the individual participants, the eligibility criteria would be people diagnosed with primary hypertension and on medical management (ICD 10 code: I10) for the last 5 years, of either gender, between the age of 18 and 60 years, who will be able to comprehend health messages in English or Kannada and have an access smartphone. We will be excluding people undergoing any other structured behavior change intervention and who are dependent for self-care.

The sample size was calculated based on the change in blood pressure status, which is the primary outcome variable, and was determined using the formula:

$$n=\frac{{\left(z_{\alpha /2}+z_{1-\beta }\right)}^{2}\left[P_{1}\left(1-P_{1}\right)+P_{2}\left(1-P_{2}\right)\right]\left[1+\left(m-1\right)\rho \right]}{{\left(P_{1}-P_{2}\right)}^{2}}$$

Where,

*n* = number of subjects in each arm of the trial

*P*_1,_ *P*_2_ = success rates in the intervention and control groups respectively

*m* = cluster size (For equal cluster size)

*ρ* = Intraclass correlation coefficient

1+( *m-*1 *) ρ* = Design effect

Considering a design effect of 1.45 (Intra class correlation coefficient for self-care in hypertension has been computed as 0.05 from literature) ( Lee *et al.*, 2020) and anticipating a 10% reduction in blood pressure readings attributable to the intervention, for a power of 90% at 5% level of significance for a 2-sided test, the required minimum sample in each arm is 118 distributed across 12 clusters of size 10. Therefore, the total sample size will be 236.

Randomization will be carried out at the cluster level i.e. the villages would be the units of randomization. The entire process of randomization will be carried out by non-participating biostatistics faculty. Sequence generation will be done according to the block technique, with a block size of 4 for 2 allocation categories: 'A' for the new intervention and 'B' for the standard intervention, yielding 6 different combinations or sequences. Allocation concealment will be achieved through coded opaque sealed envelopes. Thereafter, the interventions will be allocated to the recruited clusters by the investigators strictly according to the sequence of the block. Each of the 3 steps of randomization, namely, sequence generation, allocation concealment, and implementation will be carried out by an independent faculty.

**Figure 4.**
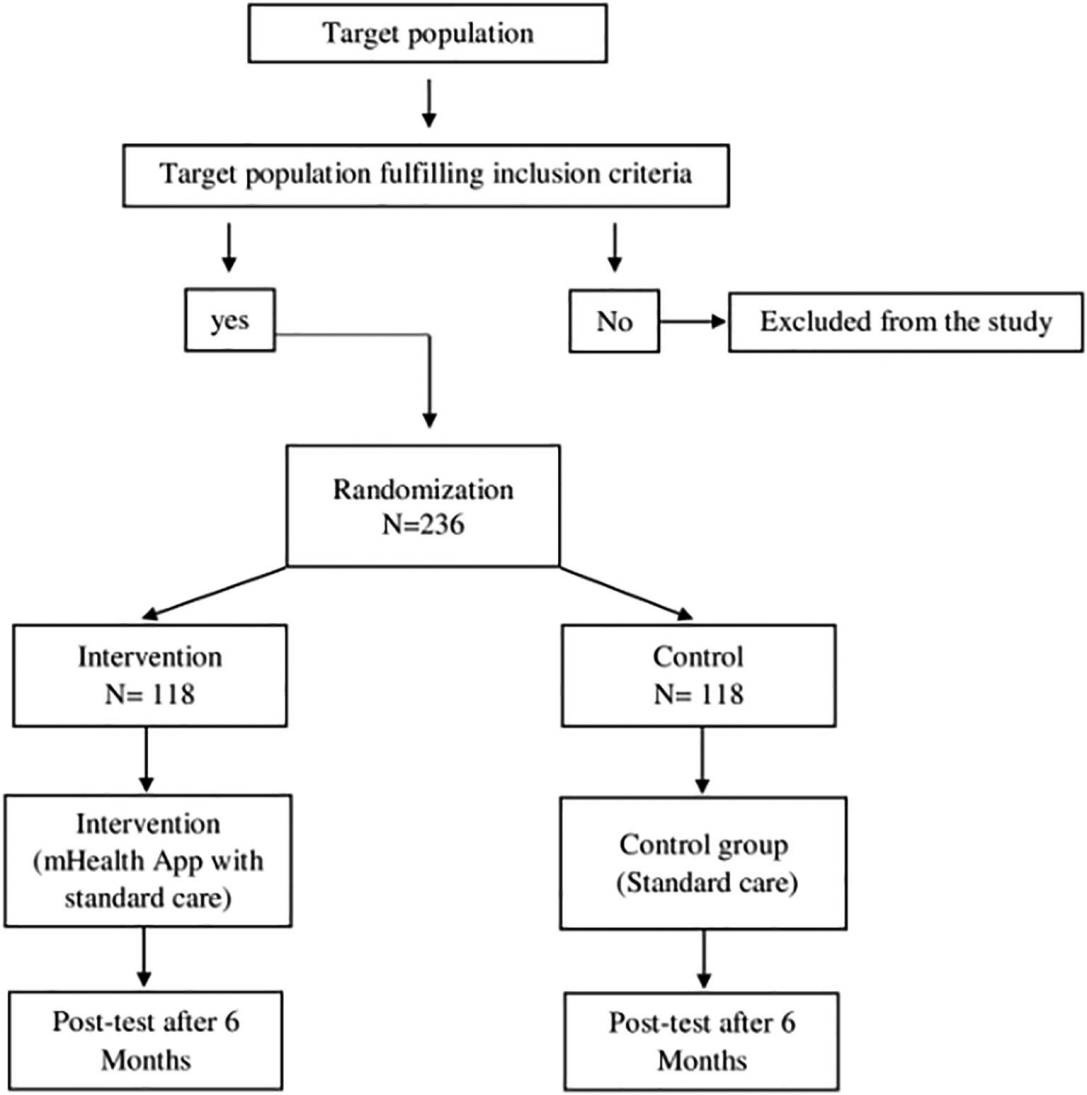
Phase 3 CONSORT flow diagram.

For the participants in the intervention group, the mHealth application will be installed on their smartphones, and the required data will be entered by the primary investigator. A demo of the application features will be provided. Participants from the intervention group will also be given automated BP apparatus and will be oriented on measuring blood pressure and the measurements will be updated in the mHealth application on the daily basis. The control group will receive standard care which includes advice to adhere to their prescribed medication and lead an active lifestyle. Baseline measurements like blood pressure, knowledge and practice of management of blood pressure, self-efficacy, and health status will be obtained from the recruited participants in both groups. Blood pressure will be measured with an electronic BP apparatus (Dr. Trust BP monitor-118), which will be calibrated periodically. Knowledge and practice will be assessed using a hypertension fact questionnaire. Self-efficacy using Medication Adherence Self-Efficacy Scale (MASES) and Self-Efficacy for Managing Chronic Disease 6-item Scale will be assessed and health status using SF-36 questionnaire will be assessed for pre and post-test ( Fernandez *et al.*, 2008; Lorig *et al.*, 2001; Ware & Gandek, 1998). For any reason, if the participant is unable to use the application, we will be discontinuing the intervention regimen for that participant. Regular phone calls will be made to remind them to use the application and to track their BP. The participants will be allowed to withdraw from the study at any given time as per their decision. The consort flowchart of the phase 3 is shown in Figure 4.

Data will be entered and analyzed using SPSS version 22. The outcomes will be analyzed at the cluster and the individual level by intention-to-treat as well as per protocol analyses. Baseline data of all collected variables will be reported at both the individual as well as at the cluster level. The flow of participants and clusters through the various stages of the trial will be depicted through a flow diagram, giving the absolute number and reasons for non-inclusion, and non-adherence at various steps from the point of approach, recruitment, randomization, a follow-up to analysis, in both the arms. The effectiveness will be analyzed according to the principles of both intention-to-treat as well as per-protocol analysis.

*Cluster-level analysis*

Categorical data will be summarized as proportions and quantitative data as means (or medians) and standard deviations (or interquartile range). Risk will be estimated as Relative Risk (RR). Chi-square test, repeated measures ANOVA (or Friedman's Test) will be used to compare the 2 groups and assess the significance of any difference therein. An estimate of the effect size for the various variables will be reported along with its precision as a 2-sided 95% confidence interval. A p-value of less than 0.05 will be taken as statistically significant. Regression models will be used according to standard protocols to find out significant factors affecting the intervention at the cluster level.

*Individual-level analysis*

It will be similar to that of the cluster-level analysis except for the fact that adjustments will be made for clustering. Hierarchical regression will be used according to standard protocols to find out significant factors affecting the intervention. In addition, the values of the intra-cluster correlation coefficient for the various outcome measures will also be reported. Subgroup analyses will be carried out and a multiplicity of analyses will be addressed.

**Study status**

Currently the focus group discussion and the in-depth interview is on-going. We plan to complete the analysis of Phase 1 by December 2022. Phase 2 of the study which is the application development will be commenced January 2023 and completed by September 2023. Phase 3 of the study which will be the determination of the effectiveness of the application will be conducted from October 2023 to March 2025.

**Dissemination**

We will be disseminating the results of the study in the form of conference presentations and as manuscripts.

## Conclusion

Use of mHealth applications for remote monitoring and self-management helps reduce the burden on health-service system. In the proposed research, we will be developing an mHealth application for people with hypertension to meet the local health information needs, provide information in regional language and will be designed based on patient usability feedback. The application will monitor blood pressure, physical activities, medication, diet, and provide recent updates on hypertension management. This application, if found effective, can improve the health status, knowledge, and self-care approach among hypertensive patients by installing the mHealth application. Additionally, it may even minimize the problems caused for accessing healthcare due to the recent pandemic and can be the solution to evade in-person sessions.

## Authors’ contributions

All authors contributed to the ideas in this protocol. P.L.S., led the writing, and all authors approved the final version.

## Data Availability

No underlying data are associated with this article. figshare: FGD guide. https://doi.org/10.6084/m9.figshare.21463152.v1 ( Salins, 2022a). figshare: Interview guide.docx. https://doi.org/10.6084/m9.figshare.21485901.v1 ( Salins, 2022b). Data are available under the terms of the Creative Commons Attribution 4.0 International license (CC-BY 4.0). figshare: SPIRIT checklist for ‘Effectiveness of an mHealth application on remote monitoring and self-management of persons with hypertension in a coastal taluk of Udupi district: A study protocol’. https://doi.org/10.6084/m9.figshare.21485946.v1 ( Salins, 2022c).
