# Effectiveness of a mHealth application on remote monitoring and self-management of persons with hypertension in a coastal taluk of Udupi district: A study protocol for a community-based Cluster Randomized trial

**DOI:** 10.12688/f1000research.127131.2

**Published:** 2025-03-21

**Authors:** Prajwal L Salins, Suma Nair, Poornima P Kundapur, Akhilesh K Pandey, Bhageerathy Reshmi, Sabu K Mandapam

**Affiliations:** 1 Health Information Management, Manipal College of Health Professions, Manipal Academy of Higher Education, Manipal, Karnataka, 576104, India; 2 School of Public Health, DY Patil University, Navi Mumbai, Maharashtra, 400706, India; 3 Department of Data Science and Computer Applications, Manipal Institute of Technology, Manipal Academy of Higher Education, Manipal, Karnataka, 576104, India; 4 Department of Community Medicine, Kasturba Medical College, Manipal Academy of Higher Education, Manipal, Karnataka, 576104, India

**Keywords:** mHealth, remote monitoring, self-management, hypertension

## Abstract

**Background:**

Hypertension is a significant risk factor for cardiovascular disease, contributing to global mortality and disability. Approximately 30% of Indian adults are diagnosed with hypertension. Evidence supports that self-monitoring and blood pressure self-management can lower systolic BP by an average of 3.2 mmHg. mHealth applications facilitate remote monitoring and self-management, yet existing applications in India lack customisation for user needs, limiting their usability. This study aims to develop and evaluate a novel, user-friendly mHealth application tailored to hypertensive individuals.

**Methods:**

This study follows an Agile development design, an iterative software development approach that allows continuous feedback and refinement. The research will be conducted in three phases over an anticipated duration of 27 months. Phase 1 (6 months) will involve in-depth interviews and focus group discussions to identify essential features for a customised Android-based mHealth application. Phase 2 (9 months) will involve developing the application using Android Studio following Agile principles. Phase 3 (12 months) will be a community-based cluster randomised trial conducted in 12 villages to evaluate the application’s effectiveness. Villages will be randomised into intervention and control groups. Each group will include 118 participants. The intervention group will use the mHealth application, while the control group will follow the standard hypertension management regimen. Villages and participants will be selected based on specific criteria, including population size, availability of healthcare facilities, and smartphone accessibility among hypertensive patients.

**Results:**

In the proposed study, if the intervention is helpful, hypertension patients in the community can be encouraged to use the mHealth application. If found effective, this application is anticipated to improve hypertensive patients’ health status, knowledge, and self-care approach.

Registration: Clinical Trials Registry - India **( CTRI/2022/03/041544).**

## Introduction

Hypertension is a significant public health concern worldwide, with an increasing prevalence in both urban and rural communities. It is a leading cause of cardiovascular diseases such as myocardial infarction, stroke, and kidney disease, contributing significantly to global mortality and morbidity. In India, recent estimates suggest that hypertension affects between 15% and 35% of the population, with poor awareness, treatment, and control rates despite its high prevalence ( Gupta et al., 2024). A study by Ramakrishnan et al. (2019) reported that nearly 50% of hypertensive individuals in India are unaware of their condition, and only one in ten has their blood pressure under control. This highlights the urgent need for effective interventions to improve hypertension management in the country.

Managing hypertension requires a combination of lifestyle modifications, regular blood pressure monitoring, and medication adherence. Research has demonstrated that self-monitoring blood pressure can reduce systolic BP by 2–8 mmHg ( Chobanian et al., 2003; Tucker et al., 2017). However, traditional healthcare systems often fail to provide adequate support for self-management, particularly in resource-limited settings. In this context, mobile health (mHealth) applications have emerged as a promising tool to support patients in managing their conditions. mHealth refers to using mobile devices such as smartphones and tablets to provide healthcare services remotely ( Santo & Redfern, 2019). These applications can integrate self-monitoring tools (e.g., blood pressure tracking, step counting), alerts and reminders (e.g., medication adherence, follow-up visits), personalised health information (e.g., diet plans, educational materials), and feedback from healthcare providers to enhance self-care and disease management ( Alessa et al., 2018).

Despite the increasing adoption of mHealth applications, most existing solutions in India lack customisation to the local population’s needs. Many available apps are designed for Western healthcare settings, leading to usability and accessibility challenges for Indian users ( Ganeshkumar et al., 2024), such as language barriers, limited integration with regional healthcare systems, lack of user-centred design, and affordability concerns. Due to these gaps, many hypertensive patients struggle with self-monitoring and adherence to treatment regimens, leading to poor disease control and increased risk of complications. There is a pressing need for an affordable, user-friendly, and culturally appropriate mHealth application that caters to the Indian population.

To address these challenges, this study aims to develop and evaluate a customised mHealth application designed explicitly for hypertensive individuals in India. The proposed application will include Multi-language support, Blood pressure monitoring, Medication reminders, Physical activity tracking, Diet and lifestyle recommendations, Graphical progress reports, and Health alerts and emergency notifications.

Given the growing burden of hypertension in India and the limitations of existing mHealth solutions, a customised, locally relevant, and patient-centric application can improve disease self-management, enhance patient engagement, and ultimately contribute to better hypertension control. This study will develop and test a novel mHealth intervention to assess its effectiveness in promoting self-monitoring, adherence, and improved health outcomes among hypertensive individuals in a community-based setting.

## Methods

### Ethics and registration

The approval has been obtained from Kasturba Medical College and Kasturba Hospital Institutional Ethics Committee (IEC No: 587-2021), and written consent from the participants will also be obtained. The study has been registered in Clinical Trial Registry - India ( CTRI/2022/03/041544).

### Phase 1

The study is proposed to be conducted in three phases. The first phase of this study will be a qualitative one, to identify the needs and expectations of people with hypertension in the Udupi district, Southern India. This phase will be carried out in three steps:

1. Focus group discussions (FGDs) with persons diagnosed with primary hypertension
2. In-depth interviews with general physicians and cardiologists
3. Development of educational material

*Focus group discussions*

For the FGDs, we will be including persons diagnosed with primary hypertension and who are being medically managed (ICD 10 code: I10), between the age of 18 and 60, of either gender, and have access to smartphones (Android) with internet connectivity. The exclusion criteria are people with visual impairment, who cannot read and comprehend either English or Kannada, and who are dependent on self-care. We will also be including caregivers of hypertensive patients above 18 years of age, who have had experience for more than a year and can understand English or Kannada, and have access to Android-based smartphones with internet connectivity. Caregivers play a significant role in ensuring adequate patient care in Low Middle-Income Countries ( Nath SD et al., 2023). For this FGD, we will have a sample size of 8 participants. This is as per previous studies.

**Figure 1.**
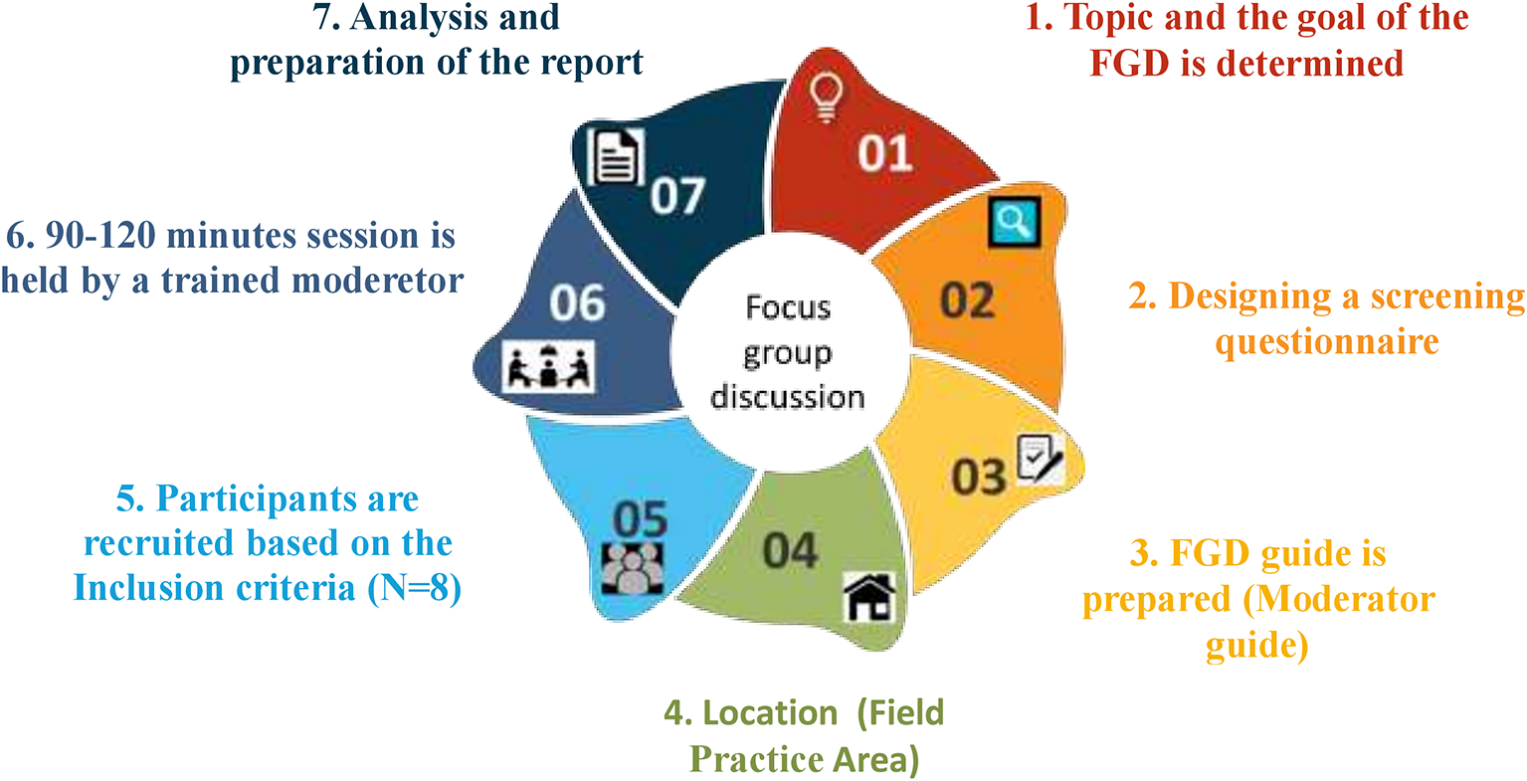
The process of focus group discussions.

The participants will be screened and recruited from health centers of Udupi district, Southern India. They will be contacted in person by the primary investigator and the written informed consent will be obtained from eligible participants after explaining the study. The recruited participants will be asked to take part in FGDs which will be carried out for the proposed objective. The moderator, who is the primary investigator, and the assistant will be introduced to the participants and the purpose of the FGD, as well as the guidelines, will be briefed to them. Before the FGDs, the screening questionnaire will be designed based on the objective of the study and validated by five experts in the field of community medicine and qualitative study experts. Subsequently, a moderator guide will be designed which includes the research rules, explanation of confidentiality, introductions to the FGD, questions, probes and follow-up questions, and conclusion. The FGDs will be audio recorded and conducted in the community health center till thematic saturation is achieved. We will be conducting thematic analysis using ATLAS.ti software version 9 and the report will be prepared. The process of the FGD has been depicted in Figure 1.

*In-depth interviews*

**Figure 2.**
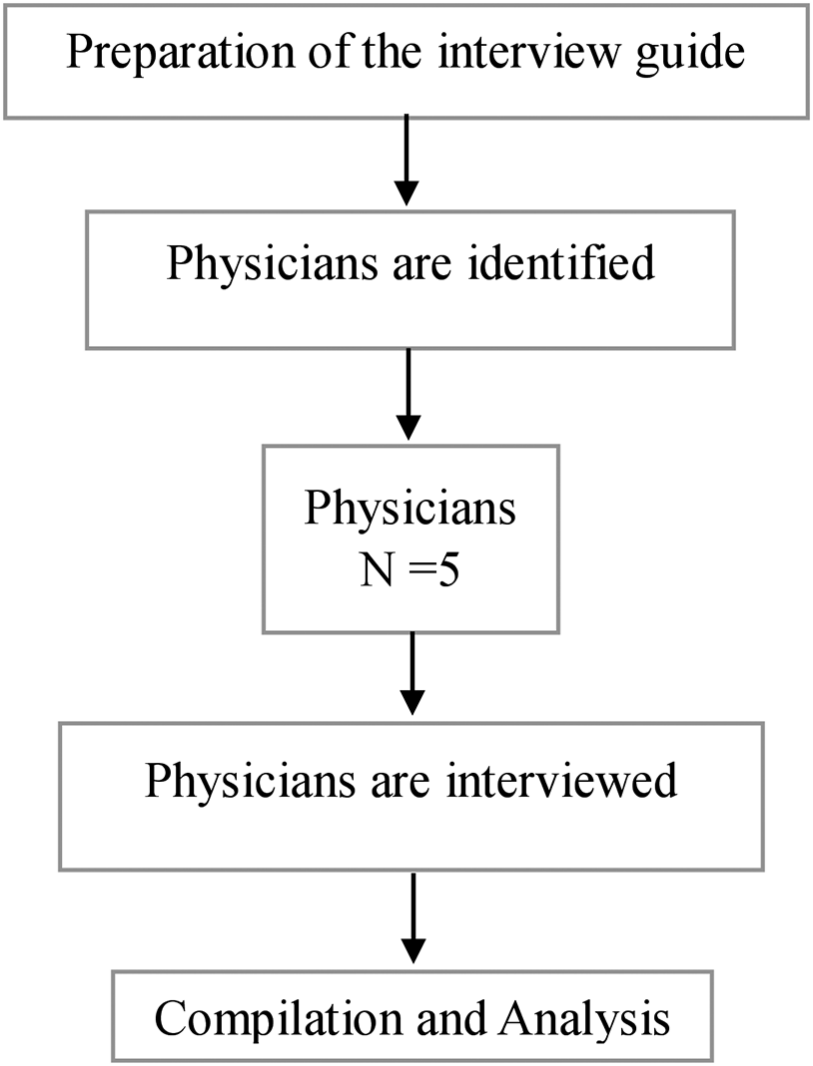
The process of in-depth interviews.

An in-depth interview guide will be prepared separately for physicians and an interview will be conducted to know their views, ideas, and opinions on the proposed mHealth application. A total of five physicians will be approached in person for the in-depth interview. Interviews will be conducted in the OPD by the primary investigator. The interview will be audio recorded. Thematic analysis will be carried out using ATLAS Ti version 9. The obtained information will be compiled and will be incorporated into the mHealth application. The process of in-depth interview is shown in Figure 2.

*Development of educational information module on self-management for persons with hypertension*

Educational information material will be prepared based on the literature review and according to recommendations for the conception and efficacy of educational tools referring to content, language, organization, layout, illustration, learning, and motivation. The developed educational information material will be translated and back-translated to and from Kannada. The information will be content reviewed using PEMAT-A/V by five physicians and five health information professionals and any comments will be considered while preparing the final version ( Shoemaker *et al.*, 2014).

### Phase 2

This phase of the study will be to develop a mHealth application for self and remote monitoring and self-management of hypertension and pilot test the application (to assess the acceptability, feasibility, usability, and user-friendliness of the app). Agile development design will be used to develop the application using Android studio ( Moe *et al.*, 2010).

Agile Software Development (ASD) has become the predominant development approach globally. ASD is used due to its beneficial features, including easy management and the ability to readily accommodate changes ( Santos et al., 2021). Agile development is a flexible approach that allows for responsiveness to changing requirements and the capacity to adapt through incremental and iterative design and feedback processes ( Tsangaris, E et al., 2022).

The mHealth application will be developed using the following ASD steps:

*Requirements analysis*

Information based on end users’ needs and expectations about the mHealth application will be captured as per the requirements to develop the mHealth application (data will be captured in phase 1 of the study).

*Designing the requirements*

Team members (i.e. Health Information Professionals, Physicians, Cardiologists, and Software developers) will be identified and the requirements of the end users will be discussed. Based on the requirements, high and low-level designing of the mHealth application will be done.

*Development*

The mHealth application will be developed based on the requirements specified by the end users. This step includes coding the application based on the design. Android Studio, an open source for Android software development will be used to develop the proposed mHealth application. The mHealth mobile application will connect to a backend database application (web-based) to help administration of content, visualization, and analysis of the data collected.

*Testing and quality assurance*

The developed application will undergo standard testing and a quality assurance process to identify and rectify the issues and bugs.

*Pilot testing and feedback*

A pilot test will be carried out once the application is developed and will be installed in Android-based smartphones among persons with hypertension to assess the acceptability, feasibility, usability, and user-friendliness of the application. The pilot phase will only include participants from the intervention group. A subset of users (n = 20-30) will test the application for usability, feasibility, and user-friendliness. Feedback from the end-users about the mHealth application would be obtained for necessary modifications in the application.

*Deployment*

The developed application will be ready for use by persons with hypertension after pilot testing for acceptance and use for self-monitoring and self-management of hypertension.

**Figure 3.**
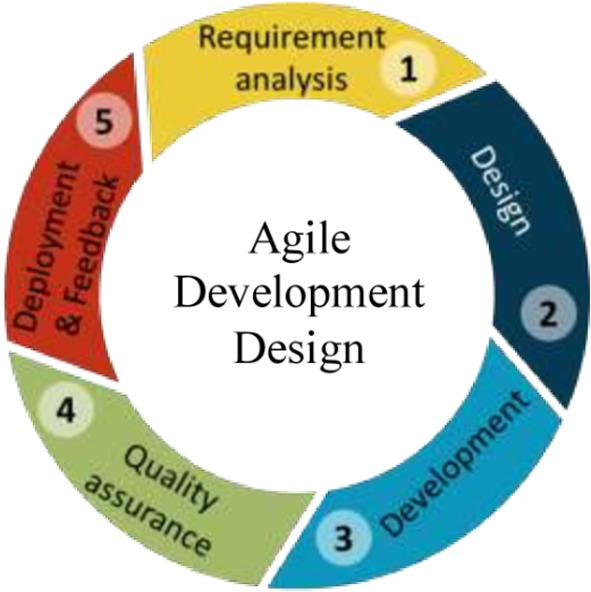
The process of Android software development.

The steps for the development of the software is shown in Figure 3.

Figure 3 shows the process of android software development.

Finally, requirement analysis will be carried out by the investigator along with experts by obtaining the requirements from the end-users. Based on the requirement, an investigator will design the mHealth application which includes various modules, layouts, tabs, widgets, and contents. The coding of the application will be done by the software developer with the help of an investigator. The developed application will be tested for quality assurance and will be pilot tested among the participants. The investigator will be involved in the front and backend process of software development. This process will take place continuously from January to September 2023.

The application will consist of blood pressure monitoring (Bluetooth enabled as well manually entered), weight, height, body mass index (BMI), medication reminder and physical activity (step count), and graphical reports of BP and weight. Weekly and monthly summaries (which can be converted into PDF and shared), abnormal warnings, Dietary Approaches to Stop Hypertension (DASH) diet plan, health education material, and recent updates on hypertension management will also be provided.

### Phase 3

An open-label, parallel cluster randomised trial will be conducted from October 2023 to March 2025, with villages as the unit of randomisation into the intervention and control arm with a 1:1 allocation. Twelve villages in the Udupi district will be considered clusters in the sampling frame and eligible for randomisation. Six villages each will be allocated to either an intervention or control group, with 118 participants in each group. Randomisation will be performed by an independent biostatistics faculty using a block randomisation method (block size = 4). Allocation will be concealed using opaque, sealed envelopes to minimise selection bias. People diagnosed with hypertension within these clusters will be the targeted population. The eligibility criteria for the clusters include a hypertensive population strength ≥ 10. If a cluster fails to have the prescribed strength, it will be clubbed with an adjacent cluster to achieve the required number. For the individual participants, the eligibility criteria would be people diagnosed with primary hypertension and on medical management (ICD 10 code: I10) for the last 5 years, of either gender, between the ages of 18 and 60 years, who will be able to comprehend health messages in English or Kannada and have an access smartphone. We will be excluding people undergoing any other structured behaviour change intervention who are dependent on self-care.

The sample size was calculated based on the change in blood pressure status, which is the primary outcome variable, and was determined using the formula:

$$n=\frac{{\left(z_{\alpha /2}+z_{1-\beta }\right)}^{2}\left[P_{1}\left(1-P_{1}\right)+P_{2}\left(1-P_{2}\right)\right]\left[1+\left(m-1\right)\rho \right]}{{\left(P_{1}-P_{2}\right)}^{2}}$$

Where,

*n* = number of subjects in each arm of the trial

*P*_1,_ *P*_2_ = success rates in the intervention and control groups respectively

*m* = cluster size (For equal cluster size)

*ρ* = Intraclass correlation coefficient

1+( *m- *1 *) ρ* = Design effect

Considering a design effect of 1.45 (Intra class correlation coefficient for self-care in hypertension has been computed as 0.05 from literature) ( Lee *et al.*, 2020) and anticipating a 10% reduction in blood pressure readings attributable to the intervention, for a power of 90% at 5% level of significance for a 2-sided test, the required minimum sample in each arm is 118 distributed across 12 clusters of size 10. Therefore, the total sample size will be 236.

Randomization will be carried out at the cluster level i.e. the villages would be the units of randomization. The entire process of randomization will be carried out by non-participating biostatistics faculty. Sequence generation will be done according to the block technique, with a block size of 4 for 2 allocation categories: ‘A’ for the new intervention and ‘B’ for the standard intervention, yielding 6 different combinations or sequences. Allocation concealment will be achieved through coded opaque sealed envelopes. Thereafter, the interventions will be allocated to the recruited clusters by the investigators strictly according to the sequence of the block. Each of the 3 steps of randomization, namely, sequence generation, allocation concealment, and implementation will be carried out by an independent faculty.

**Figure 4.**
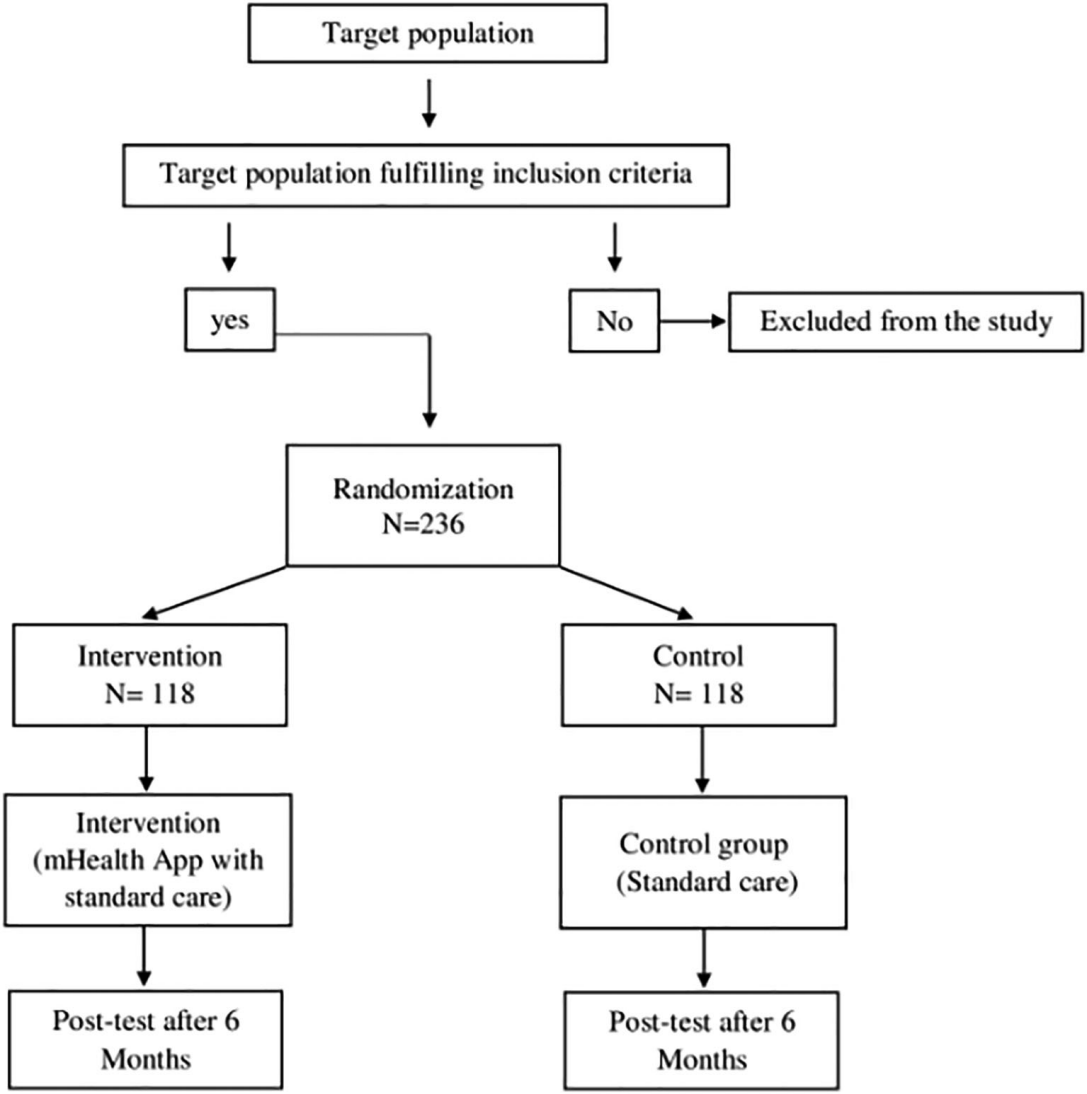
Phase 3 CONSORT flow diagram.

For the participants in the intervention group, the mHealth application will be installed on their smartphones, and the required data will be entered by the primary investigator. A demo of the application features will be provided. Participants from the intervention group will also be given automated BP apparatus and will be oriented on measuring blood pressure and the measurements will be updated in the mHealth application on the daily basis. The control group will receive standard care which includes advice to adhere to their prescribed medication and lead an active lifestyle. Baseline measurements like blood pressure, knowledge and practice of management of blood pressure, self-efficacy, and health status will be obtained from the recruited participants in both groups. Blood pressure will be measured with an electronic BP apparatus (Dr. Trust BP monitor-118), which will be calibrated periodically. Knowledge and practice will be assessed using a hypertension fact questionnaire. Self-efficacy using Medication Adherence Self-Efficacy Scale (MASES) and Self-Efficacy for Managing Chronic Disease 6-item Scale will be assessed and health status using SF-36 questionnaire will be assessed for pre and post-test ( Fernandez *et al.*, 2008; Lorig *et al.*, 2001; Ware & Gandek, 1998). For any reason, if the participant is unable to use the application, we will be discontinuing the intervention regimen for that participant. Regular phone calls will be made to remind them to use the application and to track their BP. The participants will be allowed to withdraw from the study at any given time as per their decision. The consort flowchart of the phase 3 is shown in Figure 4.

Data will be entered and analyzed using SPSS version 22. The outcomes will be analyzed at the cluster and the individual level by intention-to-treat as well as per protocol analyses. Baseline data of all collected variables will be reported at both the individual as well as at the cluster level. The flow of participants and clusters through the various stages of the trial will be depicted through a flow diagram, giving the absolute number and reasons for non-inclusion, and non-adherence at various steps from the point of approach, recruitment, randomization, a follow-up to analysis, in both the arms. The effectiveness will be analyzed according to the principles of both intention-to-treat as well as per-protocol analysis. Multiple Imputation techniques will be employed to address missing data, with sensitivity analyses conducted to determine the impact of missing data on results and the Last Observation Carried Forward method applied for cases with intermittent missing data in follow-up measurements.

*Cluster-level analysis*

Categorical data will be summarized as proportions and quantitative data as means (or medians) and standard deviations (or interquartile range). Risk will be estimated as Relative Risk (RR). Chi-square test, repeated measures ANOVA (or Friedman’s Test) will be used to compare the 2 groups and assess the significance of any difference therein. An estimate of the effect size for the various variables will be reported along with its precision as a 2-sided 95% confidence interval. A p-value of less than 0.05 will be taken as statistically significant. Regression models will be used according to standard protocols to find out significant factors affecting the intervention at the cluster level.

*Individual-level analysis*

It will be similar to that of the cluster-level analysis except for the fact that adjustments will be made for clustering. Hierarchical regression modelling will be employed to adjust for intracluster correlation in statistical comparisons due to participants being nested within village clusters, enabling assessment of the mHealth intervention’s impact on both individual and cluster-level outcomes while accounting for potential confounders, including age, gender, baseline blood pressure, and medication adherence. In addition, the values of the intra-cluster correlation coefficient for the various outcome measures will also be reported. Subgroup analyses will be carried out and a multiplicity of analyses will be addressed.

**Study status**

Currently the focus group discussion and the in-depth interview is on-going. We plan to complete the analysis of Phase 1 by December 2022. Phase 2 of the study which is the application development will commence from January 2023 to September 2023. Phase 3 of the study which will be the determination of the effectiveness of the application will be conducted from October 2023 to March 2025.

**Dissemination**

We will be disseminating the results of the study in the form of conference presentations and as manuscripts.

## Potential Strengths and Limitations

The community-based rural hypertension intervention features several notable strengths, including its cluster randomisation approach ensuring real-world applicability, user-centred design with continuous refinement through an Agile framework, multilingual accessibility in both English and Kannada, and comprehensive self-management tools covering BP tracking, medication reminders, lifestyle coaching, and emergency alerts.

Despite these strengths, the intervention faces potential limitations that require strategic mitigation. Adherence issues will be addressed through regular follow-up calls and push notifications to maintain engagement. Digital literacy barriers among participants will be overcome through hands-on training sessions before implementation. Loss to follow-up will be minimised through strategic incentives, such as providing blood pressure monitors to participants who demonstrate compliance with the study protocol.

## Conclusion

Using mHealth applications for remote monitoring and self-management helps reduce the burden on health-service system. In the proposed research, we will be developing an mHealth application for people with hypertension to meet the local health information needs, provide information in regional language and will be designed based on patient usability feedback. The application will monitor blood pressure, physical activities, medication, diet, and provide recent updates on hypertension management. This application, if found effective, can improve the health status, knowledge, and self-care approach among hypertensive patients by installing the mHealth application. Additionally, it may even minimize the problems caused for accessing healthcare due to the recent pandemic and can be the solution to evade in-person sessions.

## Authors’ contributions

All authors contributed to the ideas in this protocol. P.L.S., led the writing, and all authors approved the final version.

## Data Availability

No underlying data are associated with this article. figshare: FGD guide. https://doi.org/10.6084/m9.figshare.21463152.v1 ( Salins, 2022a). figshare: Interview guide.docx. https://doi.org/10.6084/m9.figshare.21485901.v1 ( Salins, 2022b). Data are available under the terms of the Creative Commons Attribution 4.0 International license (CC-BY 4.0). figshare: SPIRIT checklist for ‘Effectiveness of an mHealth application on remote monitoring and self-management of persons with hypertension in a coastal taluk of Udupi district: A study protocol’. https://doi.org/10.6084/m9.figshare.21485946.v1 ( Salins, 2022c).
