# Effectiveness of a mHealth application on remote monitoring and self-management of persons with hypertension in a coastal taluk of Udupi district: A study protocol for a community-based Cluster Randomized trial

**DOI:** 10.12688/f1000research.127131.3

**Published:** 2025-07-18

**Authors:** Prajwal L Salins, Suma Nair, Poornima P Kundapur, Akhilesh K Pandey, Bhageerathy Reshmi, Sabu K Mandapam

**Affiliations:** 1 Health Information Management, Manipal College of Health Professions, Manipal Academy of Higher Education, Manipal, Karnataka, 576104, India; 2 School of Public Health, DY Patil University, Navi Mumbai, Maharashtra, 400706, India; 3 Department of Data Science and Computer Applications, Manipal Institute of Technology, Manipal Academy of Higher Education, Manipal, Karnataka, 576104, India; 4 Department of Community Medicine, Kasturba Medical College, Manipal Academy of Higher Education, Manipal, Karnataka, 576104, India

**Keywords:** mHealth, remote monitoring, self-management, hypertension

## Abstract

**Background:**

Hypertension is a significant risk factor for cardiovascular disease, contributing to global mortality and disability. Approximately 30% of Indian adults are diagnosed with hypertension. Evidence supports that self-monitoring and blood pressure self-management can lower systolic BP by an average of 3.2 mmHg. mHealth applications facilitate remote monitoring and self-management, yet existing applications in India lack customisation for user needs, limiting their usability. This study aims to develop and evaluate a novel, user-friendly mHealth application tailored to hypertensive individuals.

**Methods:**

This study follows an Agile development design, an iterative software development approach that allows continuous feedback and refinement. The research will be conducted in three phases over an anticipated duration of 48 months. Phase 1 (12 months) will involve in-depth interviews and focus group discussions to identify essential features for a customised Android-based mHealth application. Phase 2 (12 months) will involve developing the application using Android Studio following Agile principles. Phase 3 (18 months) will be a community-based cluster randomised trial conducted in 12 villages to evaluate the application’s effectiveness. Villages will be randomised into intervention and control groups. Each group will include 118 participants. The intervention group will use the mHealth application, while the control group will follow the standard hypertension management regimen. Villages and participants will be selected based on specific criteria, including population size, availability of healthcare facilities, and smartphone accessibility among hypertensive patients.

**Results:**

In the proposed study, if the intervention is helpful, hypertension patients in the community can be encouraged to use the mHealth application. If found effective, this application is anticipated to improve hypertensive patients’ health status, knowledge, and self-care approach.

Registration: Clinical Trials Registry - India **( CTRI/2022/03/041544).**

## Introduction

Hypertension is a significant public health concern worldwide, with an increasing prevalence in both urban and rural communities. It is a leading cause of cardiovascular diseases such as myocardial infarction, stroke, and kidney disease, contributing significantly to global mortality and morbidity. In India, recent estimates suggest that hypertension affects between 15% and 35% of the population, with poor awareness, treatment, and control rates despite its high prevalence ( Gupta et al., 2024). A study by Ramakrishnan et al. (2019) reported that nearly 50% of hypertensive individuals in India are unaware of their condition, and only one in ten has their blood pressure under control. This highlights the urgent need for effective interventions to improve hypertension management in the country.

Managing hypertension requires a combination of lifestyle modifications, regular blood pressure monitoring, and medication adherence. Research has demonstrated that self-monitoring blood pressure can reduce systolic BP by 2–8 mmHg ( Chobanian et al., 2003; Tucker et al., 2017). However, traditional healthcare systems often fail to provide adequate support for self-management, particularly in resource-limited settings. In this context, mobile health (mHealth) applications have emerged as a promising tool to support patients in managing their conditions. mHealth refers to using mobile devices such as smartphones and tablets to provide healthcare services remotely ( Santo & Redfern, 2019). These applications can integrate self-monitoring tools (e.g., blood pressure tracking, step counting), alerts and reminders (e.g., medication adherence, follow-up visits), personalised health information (e.g., diet plans, educational materials), and feedback from healthcare providers to enhance self-care and disease management ( Alessa et al., 2018).

Despite the increasing adoption of mHealth applications, most existing solutions in India lack customisation to the local population’s needs. Many available apps are designed for Western healthcare settings, leading to usability and accessibility challenges for Indian users ( Ganeshkumar et al., 2024), such as language barriers, limited integration with regional healthcare systems, lack of user-centred design, and affordability concerns. Due to these gaps, many hypertensive patients struggle with self-monitoring and adherence to treatment regimens, leading to poor disease control and increased risk of complications. There is a pressing need for an affordable, user-friendly, and culturally appropriate mHealth application that caters to the Indian population.

To address these challenges, this study aims to develop and evaluate a customised mHealth application designed explicitly for hypertensive individuals in India. The proposed application will include Multi-language support, Blood pressure monitoring, Medication reminders, Physical activity tracking, Diet and lifestyle recommendations, Graphical progress reports, and Health alerts and emergency notifications.

While this study focuses on the Udupi district of Karnataka and incorporates Kannada language support, we recognise that India’s linguistic and cultural diversity presents unique challenges for nationwide mHealth implementation. Our choice of the Udupi district as the study site is strategic, as it represents a mixed urban-rural demographic typical of many Indian coastal regions, with established healthcare infrastructure and moderate smartphone penetration. The methodological framework and user-centred design principles employed in this study are designed to be adaptable across different linguistic and cultural contexts. However, direct generalizability will require validation studies in other Indian states’ languages and cultural nuances.

Given the growing burden of hypertension in India and the limitations of existing mHealth solutions, a customised, locally relevant, and patient-centric application can improve disease self-management, enhance patient engagement, and ultimately contribute to better hypertension control. This study will develop and test a novel mHealth intervention to assess its effectiveness in promoting self-monitoring, adherence, and improved health outcomes among hypertensive individuals in a community-based setting.

## Methods

### Ethics and registration

The approval has been obtained from Kasturba Medical College and Kasturba Hospital Institutional Ethics Committee (IEC No: 587-2021), and written consent from the participants will also be obtained. The study has been registered in Clinical Trial Registry - India ( CTRI/2022/03/041544).

### Phase 1

The study is proposed to be conducted in three phases. The first phase of this study will be a qualitative one, to identify the needs and expectations of people with hypertension in the Udupi district, Southern India. This phase will be carried out in three steps:

1. Focus group discussions (FGDs) with persons diagnosed with primary hypertension
2. In-depth interviews with general physicians and cardiologists
3. Development of educational material

*Focus group discussions*

For the FGDs, we will be including persons diagnosed with primary hypertension and who are being medically managed (ICD 10 code: I10), between the age of 18 and 60, of either gender, and have access to smartphones (Android) with internet connectivity. The exclusion criteria are people with visual impairment, who cannot read and comprehend either English or Kannada, and who are dependent on self-care. We will also be including caregivers of hypertensive patients above 18 years of age, who have had experience for more than a year and can understand English or Kannada, and have access to Android-based smartphones with internet connectivity. Caregivers play a significant role in ensuring adequate patient care in Low Middle-Income Countries ( Nath SD et al., 2023). We will conduct 3-5 focus group discussions, with each FGD comprising 6-8 participants, until thematic saturation is achieved. This approach, supported by qualitative research methodology ( Krueger & Casey, 2014), ensures comprehensive data collection while maintaining manageable group dynamics for meaningful discussion. The total sample size will range from 18 to 40 participants across all FGDs, with the final number determined by the point at which no new themes emerge from the data.

The decision to focus on Android-based smartphones strategically aligns with the Indian smartphone market landscape. According to recent market analysis, Android commands approximately 95% of India’s smartphone market share, even more pronounced in rural and semi-urban regions like the Udupi district ( Counterpoint Research, 2025). In Karnataka state specifically, Android penetration exceeds 90% among smartphone users, particularly in our target demographic of adults aged 18-60. This overwhelming market dominance is attributed to the wide availability of cost-effective Android devices across various price segments, making them accessible to diverse socioeconomic groups. The choice of the Android platform ensures maximum reach and usability among our target population while facilitating future scalability across similar demographic regions in India.

**Figure 1.**
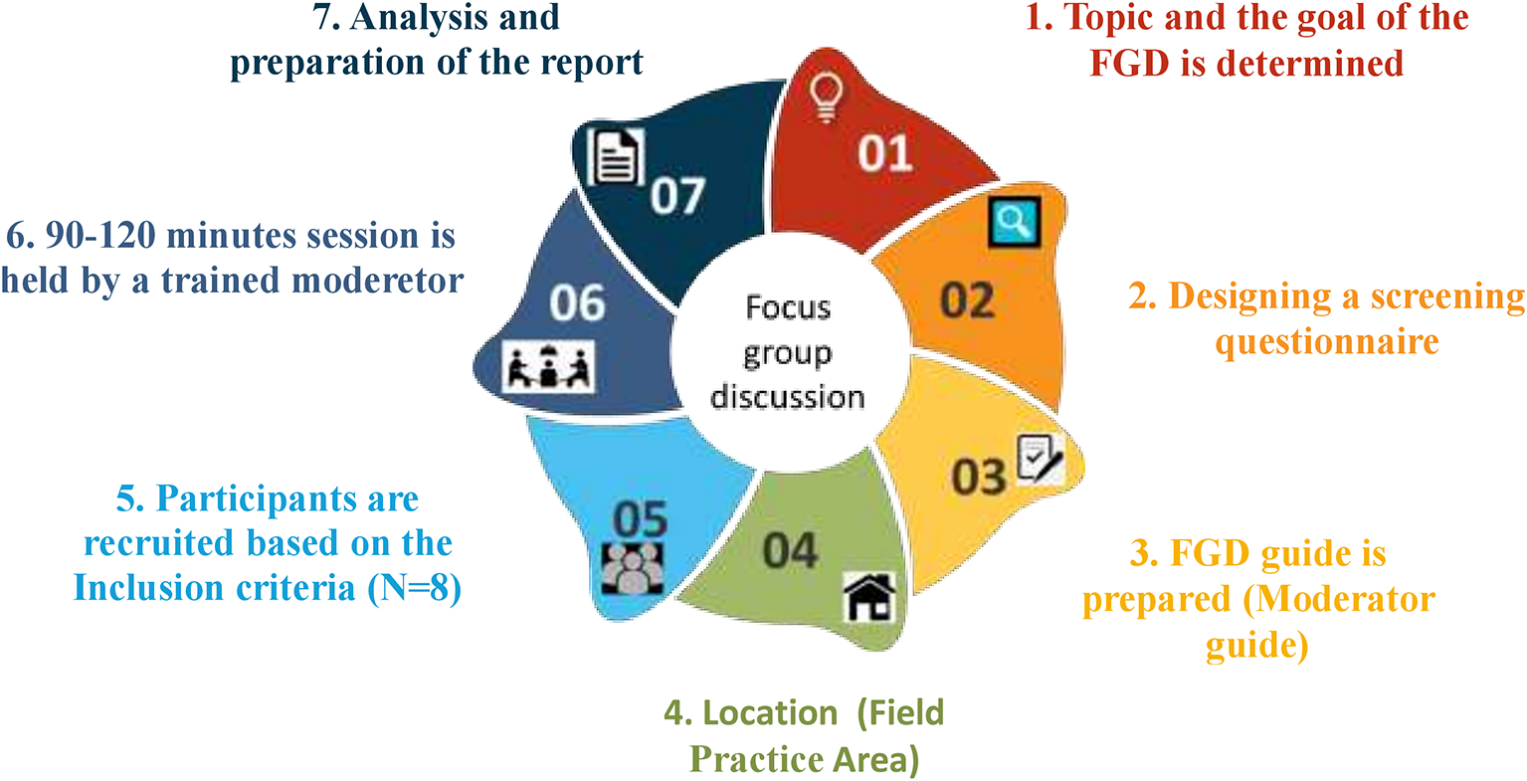
The process of focus group discussions.

The participants will be screened and recruited from health centers of Udupi district, Southern India. They will be contacted in person by the primary investigator and the written informed consent will be obtained from eligible participants after explaining the study. The recruited participants will be asked to take part in FGDs which will be carried out for the proposed objective. The moderator, who is the primary investigator, and the assistant will be introduced to the participants and the purpose of the FGD, as well as the guidelines, will be briefed to them. Before the FGDs, the screening questionnaire will be designed based on the objective of the study and validated by five experts in the field of community medicine and qualitative study experts. Subsequently, a moderator guide will be designed which includes the research rules, explanation of confidentiality, introductions to the FGD, questions, probes and follow-up questions, and conclusion. The FGDs will be audio recorded and conducted in the community health center till thematic saturation is achieved. We will be conducting thematic analysis using ATLAS.ti software version 9 and the report will be prepared. The process of the FGD has been depicted in Figure 1.

*In-depth interviews*

**Figure 2.**
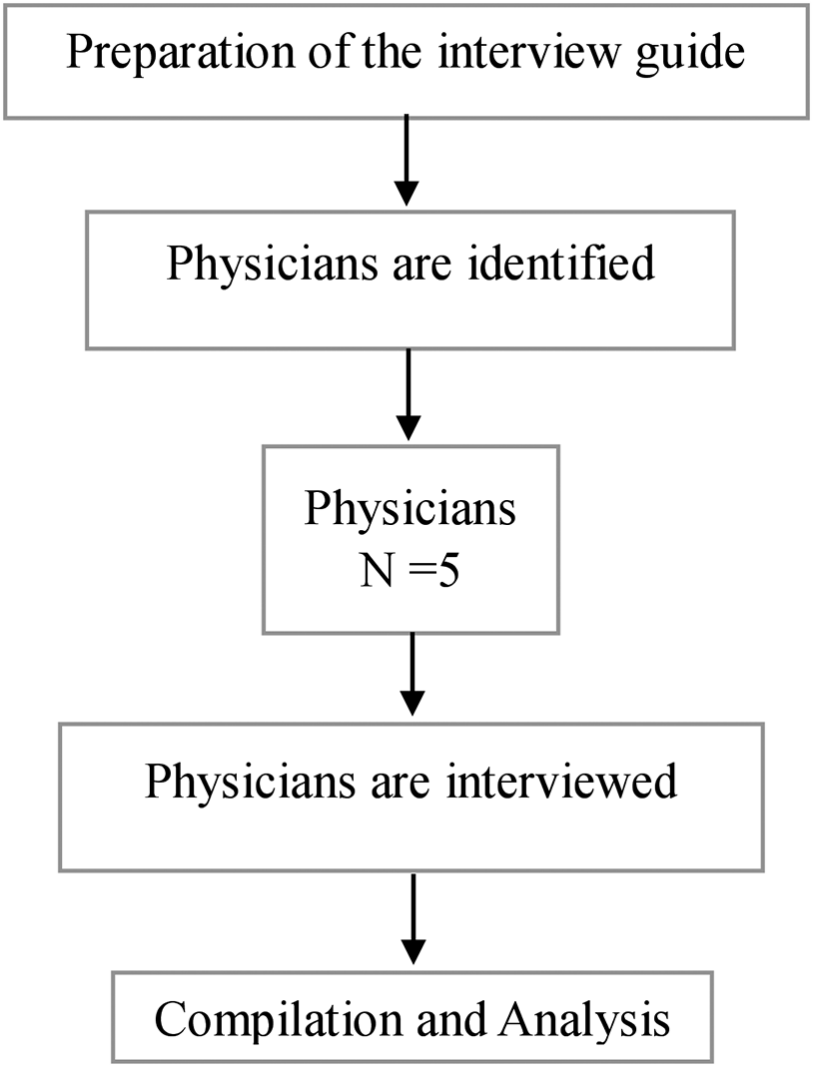
The process of in-depth interviews.

An in-depth interview guide will be prepared separately for physicians and an interview will be conducted to know their views, ideas, and opinions on the proposed mHealth application. A total of five physicians will be approached in person for the in-depth interview. Interviews will be conducted in the OPD by the primary investigator. The interview will be audio recorded. Thematic analysis will be carried out using ATLAS Ti version 9. The obtained information will be compiled and will be incorporated into the mHealth application. The process of in-depth interview is shown in Figure 2.

*Development of educational information module on self-management for persons with hypertension*

Educational materials will be developed based on a comprehensive literature review of hypertension self-management interventions and established health education design principles. Content development will follow evidence-based recommendations from the Centers for Disease Control and Prevention’s health communication guidelines ( CDC, 2014) and the Patient Education Materials Assessment Tool criteria ( Shoemaker et al., 2014), addressing six key domains: content accuracy and completeness, language clarity and cultural appropriateness, organisation and layout optimisation, visual design and illustration effectiveness, learning enhancement strategies, and motivational elements. The literature review will systematically examine peer-reviewed studies on hypertension education interventions published in the past decade, focusing on culturally adapted materials for South Asian populations. The developed educational information material will be translated and back-translated to and from Kannada. The information will be content reviewed using PEMAT-A/V by five physicians and five health information professionals and any comments will be considered while preparing the final version ( Shoemaker et al., 2014).

### Phase 2

This phase of the study will be to develop a mHealth application for self and remote monitoring and self-management of hypertension and pilot test the application (to assess the acceptability, feasibility, usability, and user-friendliness of the app). Agile development design will be used to develop the application using Android studio ( Moe et al., 2010).

Agile Software Development (ASD) has become the predominant development approach globally. ASD is used due to its beneficial features, including easy management and the ability to readily accommodate changes ( Santos et al., 2021). Agile development is a flexible approach that allows for responsiveness to changing requirements and the capacity to adapt through incremental and iterative design and feedback processes ( Tsangaris, E et al., 2022).

The mHealth application will be developed using the following ASD steps:

*Requirements analysis*

Information based on end users’ needs and expectations about the mHealth application will be captured as per the requirements to develop the mHealth application (data will be captured in phase 1 of the study).

*Designing the requirements*

Team members (i.e. Health Information Professionals, Physicians, Cardiologists, and Software developers) will be identified and the requirements of the end users will be discussed. Based on the requirements, high and low-level designing of the mHealth application will be done.

*Development*

The mHealth application will be developed based on the requirements specified by the end users. This step includes coding the application based on the design. Android Studio, an open source for Android software development will be used to develop the proposed mHealth application. The mHealth mobile application will connect to a backend database application (web-based) to help administration of content, visualization, and analysis of the data collected.

The application development process took place from January to July 2025, followed by the pilot study from August to September 2025. All feedback from the pilot study has been incorporated, and the final application version was prepared by the end of September 2025. This ensures that Phase 3 (the randomised controlled trial) begins in October 2025 with a thoroughly tested and refined intervention.

*Platform selection rationale*

Android Studio was selected as the development platform based on several factors: (1) Android’s 95% market share in the Indian smartphone market, ensuring broad accessibility among our target population; (2) The open-source nature of Android development tools, which reduces development costs and enables customisation for local needs; (3) Better integration capabilities with affordable Bluetooth-enabled blood pressure monitoring devices commonly available in the Indian market; and (4) Superior offline functionality, which is crucial given intermittent internet connectivity in rural areas of Udupi district.

*Testing and quality assurance*

The developed application will undergo standard testing and a quality assurance process to identify and rectify the issues and bugs.

*Pilot testing and feedback*

A pilot test will be carried out once the application is developed and installed on Android-based smartphones among persons with hypertension to assess the application’s acceptability, feasibility, usability, and user-friendliness. A separate pilot study will be conducted with an independent cohort of hypertensive participants (n = 20-30) who meet the same inclusion criteria as the main study but will NOT participate in the subsequent randomised controlled trial. To avoid contamination, this pilot cohort will be recruited from health centres adjacent to but separate from the 12 villages designated for the main trial. The pilot testing will assess the application’s acceptability, feasibility, usability, and user-friendliness over 4 weeks. Participants will be provided with the mHealth application and automated BP apparatus, similar to the planned intervention protocol.

Feedback from pilot participants will be collected through structured interviews, usability questionnaires, and application usage analytics. Any technical issues, user interface problems, or content gaps identified during pilot testing will be addressed through application modifications before deployment in the main trial. The pilot study will be completed entirely before recruitment commences for Phase 3, ensuring that the intervention delivered in the randomised trial represents the refined, pilot-tested version.

*Deployment*

The developed application will be ready for use by persons with hypertension after pilot testing for acceptance and use for self-monitoring and self-management of hypertension.

**Figure 3.**
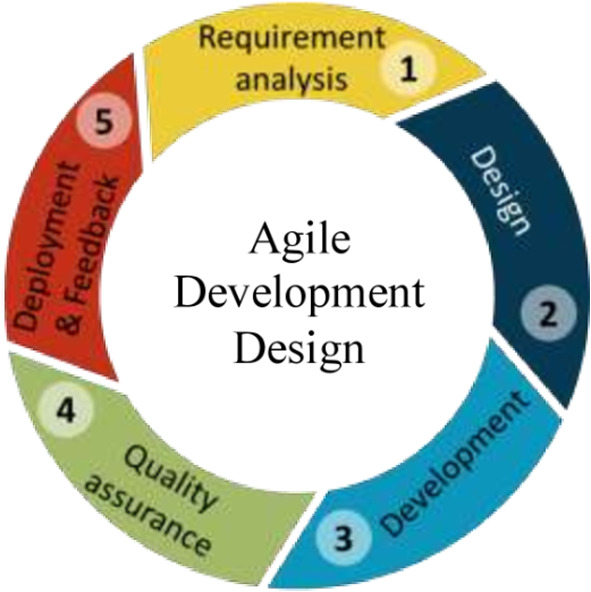
The process of Android software development.

The steps for the development of the software is shown in Figure 3.

Figure 3 shows the process of android software development.

Finally, requirement analysis will be carried out by the investigator along with experts by obtaining the requirements from the end-users. Based on the requirement, an investigator will design the mHealth application which includes various modules, layouts, tabs, widgets, and contents. The coding of the application will be done by the software developer with the help of an investigator. The developed application will be tested for quality assurance and will be pilot tested among the participants. The investigator will be involved in the front and backend process of software development. The application development process will occur from January to July 2023, followed by the pilot study from August to September 2023. All feedback from the pilot study will be incorporated, and the final application version will be prepared by the end of September 2023. This ensures that Phase 3 (the randomised controlled trial) begins in October 2023 with a thoroughly tested and refined intervention.

The application will consist of blood pressure monitoring (Bluetooth enabled as well manually entered), weight, height, body mass index (BMI), medication reminder and physical activity (step count), and graphical reports of BP and weight. Weekly and monthly summaries (which can be converted into PDF and shared), abnormal warnings, Dietary Approaches to Stop Hypertension (DASH) diet plan, health education material, and recent updates on hypertension management will also be provided.

*Emergency alert protocol and safety measures*

Emergency alerts will be triggered when systolic BP exceeds 180 mmHg or diastolic BP exceeds 110 mmHg on two consecutive readings within 24 hours, or when systolic BP falls below 90 mmHg. The alert system operates on multiple levels: (1) immediate on-screen notification advising participants to seek medical attention; (2) automated SMS to the participant’s designated emergency contact (identified during enrollment); and (3) notification to the study team’s 24/7 monitoring system, enabling direct contact with participants and their healthcare providers. To address ethical concerns, all participants will provide informed consent for this monitoring protocol, designate emergency contacts, and receive clear instructions about when to seek immediate medical care independent of app alerts.

*Recent updates on hypertension management*

The application will provide evidence-based health education content updated monthly, including: (1) medication adherence tips and side effect management; (2) lifestyle modification strategies (diet, exercise, stress management); (3) seasonal health considerations (e.g., BP management during extreme weather); and (4) simplified summaries of relevant clinical guidelines. Content will be reviewed by cardiologists and community medicine specialists before dissemination, with sources attributed and language appropriate for diverse educational backgrounds.

### Phase 3

After successfully completing the pilot study and incorporating user feedback, the refined mHealth application will be evaluated through a community-based cluster randomised controlled trial. The pilot study participants will not be included in this main trial to maintain methodological rigour and prevent contamination.

An open-label, parallel cluster randomised trial will be conducted from October 2023 to March 2025, with villages as the unit of randomisation into the intervention and control arm with a 1:1 allocation. Twelve villages in the Udupi district will be considered clusters in the sampling frame and eligible for randomisation. Six villages each will be allocated to either an intervention or control group, with 118 participants in each group. Randomisation will be performed by an independent biostatistics faculty using a block randomisation method (block size = 4). Allocation will be concealed using opaque, sealed envelopes to minimise selection bias. People diagnosed with hypertension within these clusters will be the targeted population. The eligibility criteria for the clusters include a hypertensive population strength ≥ 10. If a cluster fails to have the prescribed strength, it will be clubbed with an adjacent cluster to achieve the required number. For the individual participants, the eligibility criteria would be people diagnosed with primary hypertension and on medical management (ICD 10 code: I10) for the last 5 years, of either gender, between the ages of 18 and 60 years, who will be able to comprehend health messages in English or Kannada and have access to Android-based smartphones with internet connectivity. The Android platform requirement aligns with the prevalent smartphone ecosystem in the region, where Android devices account for over 90% of the market share among our target demographic, ensuring maximum study feasibility and real-world applicability. We will be excluding people undergoing any other structured behaviour change intervention who are dependent on self-care. Participants currently engaged in regular self-monitoring of blood pressure (defined as measuring BP ≥4 times per week for the past 3 months) will be eligible for inclusion. Still, they will be stratified during randomisation to ensure balanced distribution across intervention and control groups. This approach acknowledges that existing self-monitoring behaviour may influence intervention effectiveness while maintaining the study’s external validity.

The sample size was calculated based on the primary outcome of mean systolic blood pressure reduction, using the formula for cluster randomised trials:

$$n=\left[{\left(z_{\alpha /2}+z_{1-\beta }\right)}^{2},\left\{\left(\sigma_{1}^{2}+\sigma_{2}^{2}\right),/,\delta^{2}\right\}\right]\left[1+\left(m-1\right)\rho \right]$$

Where n = number of subjects per arm, δ = effect size, σ = standard deviation, m = cluster size, ρ = intraclass correlation coefficient.

Based on systematic review evidence demonstrating that comprehensive mHealth interventions (combining self-monitoring, medication reminders, and lifestyle support) achieve systolic BP reductions of 4-6 mmHg compared to usual care ( Mekonnen et al., 2022; Chandrasekhar et al., 2021), we anticipate a conservative effect size of 5 mmHg reduction in systolic BP. Assuming a baseline systolic BP of 145 ± 15 mmHg in our population ( Kumar et al., 2020), a five mmHg reduction represents a clinically meaningful 3.4% decrease. With 90% power, 5% significance level (two-sided), design effect of 1.45 (ICC = 0.05), and accounting for 15% attrition, the required sample size is 118 participants per arm distributed across 12 clusters of approximately 20 participants each (total n = 236). A sensitivity analysis confirms adequate power (>80%) to detect systolic BP reductions as small as 3.5 mmHg, ensuring the study can identify clinically meaningful effects consistent with published mHealth intervention studies.

Randomization will be carried out at the cluster level i.e. the villages would be the units of randomization. The entire process of randomization will be carried out by non-participating biostatistics faculty. Sequence generation will be done according to the block technique, with a block size of 4 for 2 allocation categories: ‘A’ for the new intervention and ‘B’ for the standard intervention, yielding 6 different combinations or sequences. Allocation concealment will be achieved through coded opaque sealed envelopes. Thereafter, the interventions will be allocated to the recruited clusters by the investigators strictly according to the sequence of the block. Each of the 3 steps of randomization, namely, sequence generation, allocation concealment, and implementation will be carried out by an independent faculty.

**Figure 4.**
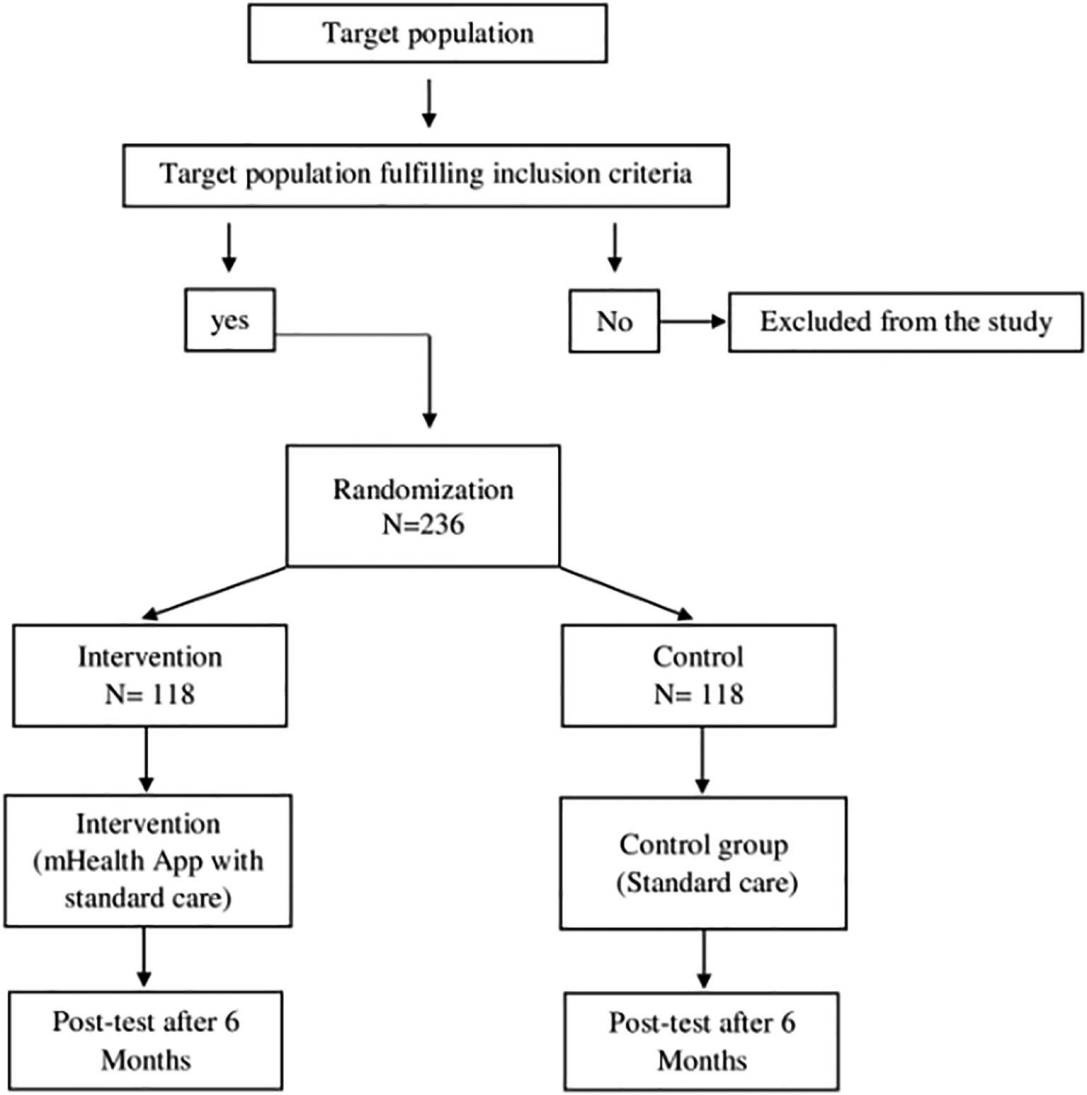
Phase 3 CONSORT flow diagram.

For the participants in the intervention group, the mHealth application will be installed on their smartphones, and the required data will be entered by the primary investigator. A demo of the application features will be provided. Participants from the intervention group will also be given automated BP apparatus. Under established clinical guidelines, participants will receive comprehensive blood pressure measurement training ( Williams et al., 2018; Whelton et al., 2018). The orientation protocol will include: (1) timing specifications requiring BP measurement twice daily (morning: 6-10 AM, evening: 6-10 PM) with at least 12 hours between readings; (2) standardised measurement technique involving three consecutive readings taken 1-2 minutes apart, with the first reading discarded and the mean of the second and third readings recorded; (3) pre-measurement requirements including 5 minutes of quiet rest, avoiding caffeine/exercise 30 minutes prior, proper cuff placement directly on skin (not over clothing), and maintaining feet flat on floor with back supported; (4) technical specifications ensuring appropriate cuff size selection and proper arm positioning at heart level; and (5) data entry protocols for recording measurements in the mHealth application immediately after each session. Participants will demonstrate competency in the BP measurement technique before the study commences, with refresher training provided at monthly follow-up contacts. The control group will receive standard care which includes advice to adhere to their prescribed medication and lead an active lifestyle. Baseline measurements like blood pressure, knowledge and practice of management of blood pressure, self-efficacy, and health status will be obtained from the recruited participants in both groups. Blood pressure will be measured with an electronic BP apparatus (Dr. Trust BP monitor-118), which will be calibrated periodically. Knowledge and practice will be assessed using a hypertension fact questionnaire. Self-efficacy using Medication Adherence Self-Efficacy Scale (MASES) and Self-Efficacy for Managing Chronic Disease 6-item Scale will be assessed and health status using SF-36 questionnaire will be assessed for pre and post-test ( Fernandez et al., 2008; Lorig et al., 2001; Ware & Gandek, 1998). For any reason, if the participant is unable to use the application, we will be discontinuing the intervention regimen for that participant. Regular phone calls will be made to remind them to use the application and to track their BP. The participants will be allowed to withdraw from the study at any given time as per their decision. The consort flowchart of the phase 3 is shown in Figure 4.

Data will be entered and analyzed using SPSS version 22. The outcomes will be analyzed at the cluster and the individual level by intention-to-treat as well as per protocol analyses. Baseline data of all collected variables will be reported at both the individual as well as at the cluster level. The flow of participants and clusters through the various stages of the trial will be depicted through a flow diagram, giving the absolute number and reasons for non-inclusion, and non-adherence at various steps from the point of approach, recruitment, randomization, a follow-up to analysis, in both the arms. The effectiveness will be analyzed according to the principles of both intention-to-treat as well as per-protocol analysis. Multiple Imputation techniques will be employed to address missing data, with sensitivity analyses conducted to determine the impact of missing data on results and the Last Observation Carried Forward method applied for cases with intermittent missing data in follow-up measurements.

*Cluster-level analysis*

Categorical data will be summarized as proportions and quantitative data as means (or medians) and standard deviations (or interquartile range). Risk will be estimated as Relative Risk (RR). Chi-square test, repeated measures ANOVA (or Friedman’s Test) will be used to compare the 2 groups and assess the significance of any difference therein. An estimate of the effect size for the various variables will be reported along with its precision as a 2-sided 95% confidence interval. A p-value of less than 0.05 will be taken as statistically significant. Regression models will be used according to standard protocols to find out significant factors affecting the intervention at the cluster level.

*Individual-level analysis*

It will be similar to that of the cluster-level analysis except for the fact that adjustments will be made for clustering. Hierarchical regression modelling will be employed to adjust for intracluster correlation in statistical comparisons due to participants being nested within village clusters, enabling assessment of the mHealth intervention’s impact on both individual and cluster-level outcomes while accounting for potential confounders, including age, gender, baseline blood pressure, and medication adherence. In addition, the values of the intra-cluster correlation coefficient for the various outcome measures will also be reported. Subgroup analyses will be carried out and a multiplicity of analyses will be addressed.

**Study status**

The focus group discussions and in-depth interviews have been completed, with Phase 1 analysis finalised in September 2022. Phase 2 of the study, including application development and pilot testing, was conducted from October 2022 to August 2023, with the pilot study completed in September-October 2023. Phase 3 of the study, which will determine the effectiveness of the refined application, is scheduled to commence in October 2023 and continue through March 2025.

**Dissemination**

We will be disseminating the results of the study in the form of conference presentations and as manuscripts.

## Potential strengths and limitations

The community-based rural hypertension intervention features several notable strengths, including its cluster randomisation approach ensuring real-world applicability, user-centred design with continuous refinement through an Agile framework, multilingual accessibility in both English and Kannada, and comprehensive self-management tools covering BP tracking, medication reminders, lifestyle coaching, and emergency alerts.

Despite these strengths, the intervention faces several significant limitations. Our inclusion criteria requiring smartphone ownership introduce significant selection bias by excluding participants who cannot afford smartphones or are less technologically literate. This bias is particularly concerning as it excludes potentially vulnerable populations who might derive the most significant benefit from accessible hypertension management tools. While smartphone penetration in Karnataka exceeds 60% overall, ownership varies substantially by socioeconomic status, age, and rural versus urban location, with rural households earning less than ₹15,000 monthly showing ownership rates of approximately 30-40%. This means our findings may not be generalisable to a substantial portion of the hypertensive population, particularly those from lower socioeconomic backgrounds. Additionally, by limiting participation to existing smartphone users, our intervention inherently selects for already technologically comfortable individuals, potentially overestimating the real-world effectiveness of mHealth interventions when deployed across diverse populations with varying digital literacy levels.

Other limitations include potential adherence issues, which will be addressed through regular follow-up calls and push notifications to maintain engagement. Loss to follow-up will be minimised through strategic incentives, such as providing blood pressure monitors to participants who demonstrate compliance with the study protocol. While this study addresses hypertension management challenges relevant across India, our findings will primarily apply to Kannada-speaking populations in coastal Karnataka. India’s remarkable linguistic diversity, varying smartphone penetration rates across states, and distinct regional healthcare practices may limit the direct application of our intervention to other regions without appropriate cultural and linguistic adaptations. However, the Agile development methodology and user-centred design principles we employ provide a replicable framework that can be adapted for other Indian states and linguistic communities.

## Conclusion

Using mHealth applications for remote monitoring and self-management helps reduce the burden on health-service system. In the proposed research, we will be developing an mHealth application for people with hypertension to meet the local health information needs, provide information in regional language and will be designed based on patient usability feedback. The application will monitor blood pressure, physical activities, medication, diet, and provide recent updates on hypertension management. This application, if found effective, can improve the health status, knowledge, and self-care approach among hypertensive patients by installing the mHealth application. Additionally, it may even minimize the problems caused for accessing healthcare due to the recent pandemic and can be the solution to evade in-person sessions.

Future research should validate this intervention across multiple Indian states, incorporating diverse languages, cultural practices, and healthcare infrastructure variations to establish broader generalizability across India’s heterogeneous population. A multi-state consortium approach could facilitate the development of a scalable mHealth platform with modular language and cultural components, ultimately contributing to nationwide hypertension management improvement. Economic evaluation studies across different socioeconomic contexts would also further inform policy decisions regarding mHealth implementation at scale.

## Authors’ contributions

All authors contributed to the ideas in this protocol. P.L.S., led the writing, and all authors approved the final version.

## Data Availability

No underlying data are associated with this article. figshare: FGD guide. https://doi.org/10.6084/m9.figshare.21463152.v1 ( Salins, 2022a). figshare: Interview guide.docx. https://doi.org/10.6084/m9.figshare.21485901.v1 ( Salins, 2022b). Data are available under the terms of the Creative Commons Attribution 4.0 International license (CC-BY 4.0). figshare: SPIRIT checklist for ‘Effectiveness of an mHealth application on remote monitoring and self-management of persons with hypertension in a coastal taluk of Udupi district: A study protocol’. https://doi.org/10.6084/m9.figshare.21485946.v1 ( Salins, 2022c).
