# Supplementary material for: Effectiveness of a mHealth application on remote monitoring and self-management of persons with hypertension in a coastal taluk of Udupi district: A study protocol for a community-based Cluster Randomized trial
Source: F1000Res. 2025 Jul 18;11:1393. Originally published 2022 Nov 28. [Version 3] doi: 10.12688/f1000research.127131.3 (PMC12579307; doi:10.12688/f1000research.127131.3)
Supplement: Peer review history

# Reviewer report: Reviewer response for version 3

**DOI:** 10.5256/f1000research.184241.r399205

**Published:** 2025-10-31

**Authors:** James A. Hodgkinson

**Affiliations:** 1 Institute of Applied Health Research, University of Birmingham, Birmingham, UK

No further comments to make - the authors have responded appropriately to my previous comments, and provided the additional requested detail.

Is the study design appropriate for the research question?

Partly

Is the rationale for, and objectives of, the study clearly described?

Partly

Are sufficient details of the methods provided to allow replication by others?

No

Are the datasets clearly presented in a useable and accessible format?

Not applicable

Reviewer Expertise:

Blood pressure self-monitoring and self-management

I confirm that I have read this submission and believe that I have an appropriate level of expertise to confirm that it is of an acceptable scientific standard.

# Reviewer report: Reviewer response for version 2

**DOI:** 10.5256/f1000research.178861.r381227

**Published:** 2025-05-26

**Authors:** James A. Hodgkinson

**Affiliations:** 1 Institute of Applied Health Research, University of Birmingham, Birmingham, UK

The rationale and objectives for the study are overall clear and important. Notwithstanding that, it would be helpful to understand if the results will be generalisable beyond the specific district of Udupi in Southern India to at least the rest of India. This is because the Introduction discusses challenges in India generally including language barriers and culturally appropriate applications; however, the study will focus on the smartphone using the Kannada language and will be undertaken in one area only of what is a very culturally diverse nation.

 The study design seems appropriate for the research question. Nevertheless, there are several aspects in the details of the methodology that require either revision or greater clarity.

 I don’t understand why the authors state “the pilot phase will only include participants from the intervention group”. The pilot phase should be quite separate from and well before participants are randomised into intervention and control groups. The point of having a pilot phase is to test what may need changing about the intervention before it is put into place. I confess I find this error somewhat troubling.

 The authors state the application will be suitable for and installed in Android-based smartphones. This may be very sensible but does need justification. Are Android phones the type most used in this area (and in India more generally)?

 Furthermore, part of the inclusion criteria is that participants have an access smartphone. This seems to me to introduce bias into the sample, as you will only be including already technically literate people in your sample. Those who cannot afford a smartphone will be excluded and this does seem inequitable as well as biased. It would be better if you supplied participants with the technology you are testing. You describe as a strength of the proposed work that “Digital literacy barriers among participants will be overcome through hands-on training sessions”, but only existing Android users are eligible for this. Perhaps Android use is so common this is not a problem, but this goes back to my previous point that you need to justify your approach by providing evidence of the extent of Android take-up in this region.

 My next concern relates to the lack of detail about the intervention. You state “participants… will be oriented on measuring blood pressure” but for this type of intervention you need to be specific. What will the orientation communicate? Will BP measurement be required both in the morning and evening, will it be specified that 3 readings are taken, with the first being ignored and the mean of readings 2-3 being used, will there be advice not to have the cuff over clothes, to rest for 5 minutes beforehand etc.? References to justify your approach are required.

 You also need to have some sort of strategy in place for potential participants who are already self-monitoring their blood pressure – will they be excluded or would they be eligible to take part?

 On the messages the app provides, I appreciate the detail will come only from the pilot phase but there are a couple of points made that could be clarified now. You say “emergency alerts” will be sent but in what circumstances exactly? If no-one but the participant sees this, does this not raise key ethical issues? You also say, “the application will… provide recent updates on hypertension management”. What does this mean?

 You provide a detailed sample size calculation, which is good to see. My concern here is why you are anticipating a 10% reduction in blood pressure. You note correctly in your abstract that evidence suggests self-monitoring and self-management can lower systolic BP by an average of 3.2mmHg (which is clinically significant and so certainly worthwhile), but this would be substantially less than 10%. On this basis, your study seems seriously underpowered.

 Finally, the paper is generally well written but there are nonetheless quite a few instances where writing and editing are a cause for concern.

 Clearly the paper has been written, reviewed and updated previously. However, errors such as specifying past dates for when particular study phases will occur should be checked before submission and will need amending. There are several of these throughout the draft.

 Furthermore, I accept the response of the authors to one point I agreed with a previous reviewer about regarding the specifics of the focus group(s). In their response, the authors say their study includes 3-5 focus groups (each with 6-8 participants), which seems sensible to me. However, this is not what is written in the submitted paper, which still gives the impression there will only be 1 focus group. This needs revising.

 The sentence “Educational information material will be prepared based on the literature review and according to recommendations for the conception and efficacy of educational tools referring to content, language, organization, layout, illustration, learning, and motivation” is not only very wordy and poorly expressed in English, but more importantly quite unclear. What literature review is being referred to here – one the authors are conducting? Where do the ‘recommendations’ come from, this needs a reference.

Is the study design appropriate for the research question?

Partly

Is the rationale for, and objectives of, the study clearly described?

Partly

Are sufficient details of the methods provided to allow replication by others?

No

Are the datasets clearly presented in a useable and accessible format?

Not applicable

Reviewer Expertise:

Blood pressure self-monitoring and self-management

I confirm that I have read this submission and believe that I have an appropriate level of expertise to state that I do not consider it to be of an acceptable scientific standard, for reasons outlined above.

# Response

**Published:** 2025-06-27

**Authors:** prajwal salins

**Affiliations:** Manipal University, India

We thank the reviewer for these detailed and constructive comments. All suggested revisions have been implemented to enhance the manuscript's clarity, methodological rigor, and clinical relevance. The updated protocol provides comprehensive details on blood pressure measurement procedures, safety protocols, realistic effect size expectations, and clear timelines, addressing all concerns raised while maintaining the study's innovative approach to mHealth intervention development and evaluation.

**Reviewer Comment:**

 The rationale and objectives for the study are overall clear and important. Notwithstanding that, it would be helpful to understand if the results will be generalisable beyond the specific district of Udupi in Southern India to at least the rest of India. This is because the Introduction discusses challenges in India generally including language barriers and culturally appropriate applications; however, the study will focus on the smartphone using the Kannada language and will be undertaken in one area only of what is a very culturally diverse nation.

**Response**

 We acknowledge that our study, being conducted in Udupi district with Kannada language support, represents a regional approach to a national problem.

 While our intervention specifically addresses the needs of Kannada-speaking populations in coastal Karnataka, we believe the methodological framework and user-centered design principles can be adapted for other regions. The Agile development approach we employ is specifically chosen to facilitate such adaptations. However, we recognize that direct generalizability is limited by linguistic, cultural, and healthcare infrastructure variations across

 Indian states.

 We have strengthened our manuscript by explicitly acknowledging these limitations in our "Potential Strengths and Limitations" section and have added recommendations for future multi-state validation studies in our conclusion. This transparency regarding scope limitations actually enhances the study's credibility while providing a clear pathway for broader implementation.

**Reviewer Comment**

 I don’t understand why the authors state “the pilot phase will only include participants from the intervention group”. The pilot phase should be quite separate from and well before participants are randomised into intervention and control groups. The point of having a pilot phase is to test what may need changing about the intervention before it is put into place. I confess I find this error somewhat troubling.

**Response**

 We have revised our methodology to clarify that:

1. The pilot testing will be conducted with a separate cohort of participants (n=20-30) who will NOT be part of the subsequent randomized controlled trial
2. The pilot phase will be completed entirely before recruitment begins for Phase 3
3. Modifications based on pilot feedback will be implemented before the main study commences
4. The timeline has been adjusted to reflect this proper sequence

This correction ensures methodological rigor and aligns with established best practices for intervention development and testing. We appreciate the reviewer's attention to this important detail, as it significantly strengthens our study design.

**Reviewer Comment**

 The authors state the application will be suitable for and installed in Android-based smartphones. This may be very sensible but does need justification. Are Android phones the type most used in this area (and in India more generally)?

**Response**

 The decision to develop for Android is indeed strategic and evidence-based. According to recent market research data, Android holds approximately 95% of the smartphone market share in India, making it the overwhelmingly dominant platform. This dominance is particularly pronounced in rural and semi-urban areas like our study region, where cost-effective Android devices are more accessible than iOS alternatives.

 In Karnataka state specifically, Android market penetration exceeds 90%, with the majority of users in the 18-60 age demographic (our target population) using Android devices. This high market share is driven by the availability of affordable Android smartphones from various manufacturers, making them accessible across different socioeconomic strata.

 We have added appropriate justification to our manuscript with supporting references to strengthen this methodological choice.

**Reviewer Comment**

 Furthermore, part of the inclusion criteria is that participants have an access smartphone. This seems to me to introduce bias into the sample, as you will only be including already technically literate people in your sample. Those who cannot afford a smartphone will be excluded and this does seem inequitable as well as biased. It would be better if you supplied participants with the technology you are testing. You describe as a strength of the proposed work that “Digital literacy barriers among participants will be overcome through hands-on training sessions”, but only existing Android users are eligible for this. Perhaps Android use is so common this is not a problem, but this goes back to my previous point that you need to justify your approach by providing evidence of the extent of Android take-up in this region.

**Response**

 The reviewer is absolutely correct that requiring smartphone ownership introduces significant selection bias by pre-selecting participants who are technologically literate and economically advantaged enough to own smartphones. This indeed excludes potentially vulnerable populations who might benefit most from accessible hypertension management interventions.

 We acknowledge this as a substantial limitation of our study design. Due to budget constraints and logistical challenges, we are unable to provide smartphones to participants. However, we have revised our limitations section to transparently acknowledge this bias and its implications for the generalizability of our findings. We also recognize the contradiction in claiming to address digital literacy barriers while only including participants who already have smartphone access.

 Recent data from Karnataka indicates that while smartphone penetration is high in urban areas (>90%), it varies significantly by socioeconomic status and rural location. In rural areas of coastal Karnataka, smartphone ownership ranges from 40-70% depending on income levels, meaning our study will indeed exclude a substantial portion of the hypertensive population, particularly those from lower socioeconomic backgrounds.

 This transparent acknowledgment of limitations, while not ideal, strengthens the scientific integrity of our study and provides important context for interpreting results and planning future research.

**Reviewer Comment**

 My next concern relates to the lack of detail about the intervention. You state “participants… will be oriented on measuring blood pressure” but for this type of intervention you need to be specific. What will the orientation communicate? Will BP measurement be required both in the morning and evening, will it be specified that 3 readings are taken, with the first being ignored and the mean of readings 2-3 being used, will there be advice not to have the cuff over clothes, to rest for 5 minutes beforehand etc.? References to justify your approach are required.

**Response**

 We have comprehensively detailed the blood pressure measurement protocol based on current clinical practice guidelines. The protocol ensures standardized, accurate measurements while maintaining feasibility for daily self-monitoring in a community setting.

**Reviewer Comment**

 You also need to have some sort of strategy in place for potential participants who are already self-monitoring their blood pressure – will they be excluded or would they be eligible to take part?

 On the messages the app provides, I appreciate the detail will come only from the pilot phase but there are a couple of points made that could be clarified now. You say “emergency alerts” will be sent but in what circumstances exactly? If no-one but the participant sees this, does this not raise key ethical issues? You also say, “the application will… provide recent updates on hypertension management”. What does this mean?

**Response**

 We have clarified our approach to participants with existing self-monitoring practices and detailed the emergency alert protocol with appropriate safety measures and ethical considerations. The "recent updates" feature has been explicitly defined to ensure clinical relevance and safety.

**Reviewer Comment**

 You provide a detailed sample size calculation, which is good to see. My concern here is why you are anticipating a 10% reduction in blood pressure. You note correctly in your abstract that evidence suggests self-monitoring and self-management can lower systolic BP by an average of 3.2mmHg (which is clinically significant and so certainly worthwhile), but this would be substantially less than 10%. On this basis, your study seems seriously underpowered.

**Response**

 We acknowledge the reviewer's concern about our initial 10% reduction assumption. We have recalculated the sample size based on realistic effect sizes (4-6 mmHg) from recent systematic reviews of comprehensive mHealth interventions, ensuring adequate power to detect clinically meaningful blood pressure reductions while maintaining statistical rigor.

**Reviewer Comment**

 Finally, the paper is generally well written but there are nonetheless quite a few instances where writing and editing are a cause for concern.

 Clearly the paper has been written, reviewed and updated previously. However, errors such as specifying past dates for when particular study phases will occur should be checked before submission and will need amending. There are several of these throughout the draft.

**Response**

 All timeline references have been updated to reflect current study progress and realistic future timelines, ensuring consistency throughout the manuscript.

**Reviewer Comment**

 Furthermore, I accept the response of the authors to one point I agreed with a previous reviewer about regarding the specifics of the focus group(s). In their response, the authors say their study includes 3-5 focus groups (each with 6-8 participants), which seems sensible to me. However, this is not what is written in the submitted paper, which still gives the impression there will only be 1 focus group. This needs revising.

**Response**

 We have clarified that multiple focus group discussions will be conducted rather than a single FGD, providing appropriate justification for the sample size and methodology consistent with qualitative research best practices.

**Reviewer Comment**

 The sentence “Educational information material will be prepared based on the literature review and according to recommendations for the conception and efficacy of educational tools referring to content, language, organization, layout, illustration, learning, and motivation” is not only very wordy and poorly expressed in English, but more importantly quite unclear. What literature review is being referred to here – one the authors are conducting? Where do the ‘recommendations’ come from, this needs a reference.

**Response**

 We have clarified the educational material development process, specifying the literature review scope and providing appropriate references for the design recommendations. The revised text is more concise and clearly articulates the evidence-based approach to content development.

# Reviewer report: Reviewer response for version 2

**DOI:** 10.5256/f1000research.178861.r372078

**Published:** 2025-04-28

**Authors:** Kamakshi Lakshminarayan

**Affiliations:** 1 University of Minnesota Foundation, Minneapolis, Minnesota, USA

The authors have been responsive and I have no further comments. Recommend Acceptance.

Is the study design appropriate for the research question?

Partly

Is the rationale for, and objectives of, the study clearly described?

Partly

Are sufficient details of the methods provided to allow replication by others?

No

Are the datasets clearly presented in a useable and accessible format?

Not applicable

Reviewer Expertise:

NA

I confirm that I have read this submission and believe that I have an appropriate level of expertise to confirm that it is of an acceptable scientific standard.

# Reviewer report: Reviewer response for version 1

**DOI:** 10.5256/f1000research.139606.r196844

**Published:** 2024-05-31

**Authors:** Kamakshi Lakshminarayan

**Affiliations:** 1 University of Minnesota Foundation, Minneapolis, Minnesota, USA

The paper describes the protocol to develop and test a customized mHealth application to monitor HTN. The investigators describe a 3 step process including a needs and expectation assessment (phase 1), development and pilot testing a mHealth application for HTN management (phase 2), and conducting a cluster randomized trial to definitively test the mHealth application for HTN management (phase 3).

**DETAILED COMMENTS**

**Introduction**

 Improving HTN management is important since the prevalence of HTN is high (~30%).

 The authors state that the awareness of HTN, treatment and control are poor in India (para 4, line 1). However, they do not cite the numbers or references to back up this claim. Similarly, data sources or references for the fact presented that 33% of Indians use mHealth in daily life would be helpful.

**Methods**

**Phase 1**

 In phase 1, the investigators will identify needs and expectations of people with HTN in Udupi using FGDs with patients, in-depth interviews with physicians and development of educational material. Re: the FGD, they propose a sample size of 8 participants total. Does this include patients and caregivers? Does this mean that they will have a single focus group of 8 participants? Re: sample size – a focus group size ranging from 5-8 is reasonable. However, literature also supports the use of multiple focus groups to reach thematic saturation with 3-6 individual focus groups (each group has multiple participants) needed to reach 90% saturation. (Guest G et al, 2017) [Ref 1]. Will the focus groups proposed by the authors encompass both genders? Be socioeconomically diverse? Overall the Focus Groups need to be re-evaluated in terms of being representative of the patient population (sex, age, education, socioeconomic status) which is hard to do with just a sample size of 8.

**Phase 2**

 In Phase 2, the team will develop a mHealth monitoring for self-monitoring and self-management of HTN. They also state that there is remote monitoring. Remote monitoring implies that the BP data are being transmitted to someone – a healthcare provider? If the data is being transmitted, this is not explained clearly. There insufficient information on development and it is hard to evaluate the experience of the development team and whether the implementation will be successful. How many patients will evaluate the pilot implementation. The application includes BP monitoring and weight / height / BMI and step counts and medication reminder. How is weight monitored? Is it a digital scale? How often is weight measured? Are they tracking weight and BMI as outcomes in addition to BP? Is there a fitbit application to perform step count? There are too many missing details.

**Phase 3**

 While the mHealth BP app is going to be developed – it is not clear that this is necessary. The BP monitor that they propose – Dr. Trust BP monitor has an App. The team will save time and resources by using the pre-existing App and perhaps only creating an app for medication reminders, nutrition support, and physical activity measurement.

 What are all the outcomes? Is it change in systolic and diastolic BP, change in BMI as well as changes in self-efficacy? This needs to be clearly specified.

 Overall, this protocol lacks sufficient detail and specification.

Is the study design appropriate for the research question?

Partly

Is the rationale for, and objectives of, the study clearly described?

Partly

Are sufficient details of the methods provided to allow replication by others?

No

Are the datasets clearly presented in a useable and accessible format?

Not applicable

Reviewer Expertise:

Research in mHealth facilitated management of hypertension by engaging patients, post-stroke outcomes and post-stroke cognitive impairment.

I confirm that I have read this submission and believe that I have an appropriate level of expertise to state that I do not consider it to be of an acceptable scientific standard, for reasons outlined above.

## References

[1] : How Many Focus Groups Are Enough? Building an Evidence Base for Nonprobability Sample Sizes. *Field Methods* .2017;29(1) : 10.1177/1525822X16639015 3-22 10.1177/1525822X16639015

# Response

**Published:** 2025-03-06

**Authors:** prajwal salins

**Affiliations:** Manipal University, India

**Response to Reviewer Comments**

 We appreciate the reviewer’s detailed feedback and recognize the concerns raised regarding our study methodology. Below, we provide justifications for our chosen approach and clarify specific methodological decisions.

**Introduction**

**Comment:** The manuscript states that awareness, treatment, and control of hypertension (HTN) are poor in India but lacks supporting data. Similarly, the claim that **33% of Indians use mHealth applications** needs citation.

**Response:** The prevalence of **poor hypertension awareness, treatment, and control in India** has been well documented in national surveys and published studies. For example, research indicates that **nearly 50% of hypertensive individuals in India are unaware of their condition, and only 10% have controlled BP** ( **Ramakrishnan et al., 2019**). We have referenced these sources in our revised manuscript. Additionally, the **33% mHealth usage statistic** is based on a study by **Santo & Redfern (2019)**, which examines digital health adoption in India. These references support our rationale for exploring mHealth interventions in this setting.

**Phase 1 – Needs Assessment**

**Comment:** The focus group discussion (FGD) sample size of **8 participants** may not be sufficient for **thematic saturation**. The reviewer suggests multiple focus groups to ensure diversity in **gender, age, and socioeconomic status**.

**Response:** Our initial approach was based on prior qualitative studies where **small, targeted FGDs** were used to explore user needs in mHealth design. However, we acknowledge that **achieving thematic saturation requires a broader representation**. To address this, our study includes **3–5 focus groups (each with 6–8 participants)**, as supported by **Guest et al. (2017)**, ensuring a more diverse and representative sample.

 Additionally, caregivers were included because **they play a critical role in patient adherence, decision-making, and support**, particularly in low-resource settings where self-management can be challenging. Their perspectives are essential to designing a user-friendly and effective application.

**Phase 2 – Application Development**

**Comment:** The manuscript states that the mHealth app will support **remote BP monitoring**, but it is unclear if data will be transmitted to healthcare providers. Additional details on **development team expertise, pilot testing, weight tracking, and step count measurement** are needed.

**Response:** The term **“remote monitoring”** refers to **self-monitoring of BP**, with users manually entering or syncing BP data within the app. However, there will be **no automatic data transmission to healthcare providers** due to **infrastructure limitations and data privacy concerns**. Instead, users will have the option to **export and share their BP reports** with healthcare professionals.

 Our **development team** includes **health information professionals, software developers, physicians, and community health experts**, ensuring that both technological and clinical perspectives are incorporated.

 For **pilot testing**, we have chosen a **sample of 20–30 participants** to assess usability and acceptability before full-scale implementation, following established mobile health evaluation frameworks.

**Weight tracking** will involve **digital scales**, with users prompted to log weight **weekly**. **Step counts** will be measured via **smartphone sensors** rather than Fitbit integration to ensure **cost-effectiveness and accessibility** in resource-limited settings.

**Phase 3 – Cluster Randomized Trial**

**Comment:** The reviewer questions whether it is necessary to **develop a new BP monitoring application** when the **Dr. Trust BP monitor already has an app**. Would it be more efficient to **focus on other intervention components (e.g., medication reminders, nutrition, and activity tracking)?**

**Response:** While the **Dr. Trust BP monitor** has a companion app, it is **not customized for comprehensive hypertension self-management**. Our application is designed as an **integrated, multilingual platform** that combines:

- **BP monitoring** (manual entry + Bluetooth integration)
- **Medication reminders**
- **Dietary guidance (DASH-based recommendations)**
- **Step tracking**
- **Educational resources**
- **Emergency alerts for abnormal BP readings**

The **existing Dr. Trust app does not offer these features**, limiting its usefulness for long-term hypertension management. Furthermore, many BP monitor apps lack **local language support, culturally relevant content, and integration with personalized hypertension care strategies**. Our mHealth application aims to **bridge this gap** by providing a **comprehensive, patient-centered tool** for self-management.

**Outcomes and Statistical Analysis**

**Comment:** The study does not clearly define **all outcome measures** (e.g., changes in **systolic/diastolic BP, BMI, self-efficacy**).

**Response:** The primary outcome of this study is **change in systolic and diastolic BP**. Secondary outcomes include **BMI changes, self-efficacy, medication adherence, and health literacy improvements**. These measures align with prior research on digital health interventions for hypertension management and have been explicitly stated in the revised manuscript.

**Conclusion**

 We appreciate the reviewer’s concerns and have provided justifications for our methodology. Our approach ensures **cultural adaptability, patient-centered design, and rigorous evaluation** of an **integrated mHealth solution** tailored for hypertension management in India. We hope this response clarifies our methodological choices and the significance of our study.

# Reviewer report: Reviewer response for version 1

**DOI:** 10.5256/f1000research.139606.r196811

**Published:** 2023-12-12

**Authors:** Blessing Onyinye Ukoha-kalu

**Affiliations:** 1 University of Nottingham, Nottingham, England, UK

Thank you for the opportunity to review this paper. 

 I must commend the authors for taking the time to write down this protocol in a clear manner however, I have few comments which I believe will improve the clarity and reproducibility of the protocol.

 Abstract

1. The abstract is generally clear, but some sentences could be streamlined for improved readability. It is important that readers who are not in this field are able to understand how this research will be carried out.
2. To enhance the impact of your opening statement, you might add some statistics or key figures related to the global prevalence of hypertension and its impact on health.
3. In the "Methods" section, consider briefly explaining what Agile development design is, as this might not be familiar to all readers.
4. You could mention the anticipated duration of each phase, providing a rough timeline for the study.
5. Instead of stating the sample size as "236 people from 12 villages," you might specify how many participants will be assigned to each group (intervention and control) to give a clearer sense of the distribution.
6. Mention any criteria used for selecting these specific villages and participants.

**Introduction**

1. When citing statistics or findings, consider including more recent sources, especially if available, to provide up-to-date information.
2. Whenever possible, include a sentence or two explaining the findings or implications of the cited studies.
3. It might be beneficial to briefly explain what kind of self-monitoring tasks, alerts, personalized information, and feedback mHealth apps provide to readers who might not be familiar with the concept.
4. You could elaborate more on the challenges faced due to the lack of applications customized to the Indian population, including how this gap impacts hypertension management.
5. Specify the key features of the mHealth application you plan to develop. For example, will it be available in regional languages? What specific features will it offer for hypertension self-management?

**Methods**

1. Mention the name of the institution where the Institutional Ethics Committee approval was obtained.
2. The study design could be reflected in the title of the study example: randomised clinical trial, mixed-methods, qualitative e.t.c
3. When discussing FGDs, consider explaining the rationale behind involving caregivers of hypertensive patients in the discussions.
4. Provide a brief explanation of Agile development design for readers who might not be familiar with this approach.
5. Clarify whether the application development will occur continuously from January to September 2023 or if it will have distinct development periods.
6. Elaborate on the randomization process at the cluster level, including how the blocks are assigned to intervention and control arms.
7. Mention how many clusters will be in each arm after randomization.
8. Clarify whether the pilot test and feedback phase will include both intervention and control groups or only the intervention group.
9. Specify how missing data will be handled in the analysis.
10. Provide a brief explanation of hierarchical regression and its role in the analysis.
11. Kindly state the potential strenghts and limitations you may encounter while carrying out this study and how you intend to circumvent these.

**References**

 I strongly encourage the use of more recent literature and citations.

Is the study design appropriate for the research question?

Partly

Is the rationale for, and objectives of, the study clearly described?

Partly

Are sufficient details of the methods provided to allow replication by others?

Partly

Are the datasets clearly presented in a useable and accessible format?

Not applicable

Reviewer Expertise:

disease management, clinical trials, mental health, evidence synthesis, mixed methods study

I confirm that I have read this submission and believe that I have an appropriate level of expertise to confirm that it is of an acceptable scientific standard, however I have significant reservations, as outlined above.

# Response

**Published:** 2025-03-01

**Authors:** prajwal salins

**Affiliations:** Manipal University, India

We sincerely appreciate the valuable time and effort the reviewers have invested in evaluating our manuscript. Their insightful comments and constructive suggestions have improved our study’s clarity, depth, and quality. We have carefully addressed all the reviewers’ concerns and have made the necessary revisions throughout the manuscript. We are grateful for the reviewers’ thoughtful feedback, which strengthened our manuscript. We hope the revised version meets their expectations and await their continued guidance.

**Abstract**

**Reviewer comments**

**Author response**

**Page no. and Line no.**

 The abstract is generally clear, but some sentences could be streamlined for improved readability. It is important that readers who are not in this field are able to understand how this research will be carried out.

 The abstract has been revised to increase readability.

 Page no.: 1

 To enhance the impact of your opening statement, you might add some statistics or key figures related to the global prevalence of hypertension and its impact on health.

 We have added the statistics showcasing the prevalence of hypertension and its impact.

 Page no.: 1

 Line no.: 5-7

 In the "Methods" section, consider briefly explaining what Agile development design is, as this might not be familiar to all readers.

 The revised version explains about the Agile development.

 Page no.: 1

 Line no.: 12-13

 You could mention the anticipated duration of each phase, providing a rough timeline for the study.

 We have provided an estimated study timeline.

 Page no.: 1

 Line no.: 13-19

 Instead of stating the sample size as "236 people from 12 villages," you might specify how many participants will be assigned to each group (intervention and control) to give a clearer sense of the distribution.

 We have specified the participant distribution in the trial.

 Page no.: 1

 Line no.: 19-20

 Mention any criteria used for selecting these specific villages and participants.

 We have included the selection criteria for villages and participants.

 Page no.: 1

 Line no.: 21-23

**Introduction**

 When citing statistics or findings, consider including more recent sources, especially if available, to provide up-to-date information.

 We have updated statistics with recent references

 Page no.: 2-3

 Whenever possible, include a sentence or two explaining the findings or implications of the cited studies.

 We have explained implications of hypertension prevalence studies.

 Page no.: 2

 Line no.: 39-46

 It might be beneficial to briefly explain what kind of self-monitoring tasks, alerts, personalized information, and feedback mHealth apps provide to readers who might not be familiar with the concept.

 We have provided with detailed explanation of mHealth functionalities.

 Page no.: 2

 Line no.: 46-50

 You could elaborate more on the challenges faced due to the lack of applications customized to the Indian population, including how this gap impacts hypertension management.

 We have expanded discussion on limitations of existing mHealth apps in India.

 Page no.: 3

 Line no.: 51-59

 Specify the key features of the mHealth application you plan to develop. For example, will it be available in regional languages? What specific features will it offer for hypertension self-management?

 We have clearly specified the features of the proposed app.

 Page no.: 3

 Line no.: 61-64

**Methods**

 Mention the name of the institution where the Institutional Ethics Committee approval was obtained.

 We have mentioned the name of the institution

 Page no.: 3

 Line no.: 73-74

 The study design could be reflected in the title of the study example: randomised clinical trial, mixed-methods, qualitative e.t.c

 We have revised the title according the suggestion

 Page no.: 1

 Line no.: 1-3

 When discussing FGDs, consider explaining the rationale behind involving caregivers of hypertensive patients in the discussions.

 We have clarified the role of caregivers in FGDs

 Page no.: 4

 Line no.: 91-93

 Provide a brief explanation of Agile development design for readers who might not be familiar with this approach.

 We have provided a brief explanation of Agile development design.

 Page no.: 6

 Line no.: 135-139

 Clarify whether the application development will occur continuously from January to September 2023 or if it will have distinct development periods.

 Clarified.

 Page no.: 8

 Line no.: 177-178

 Elaborate on the randomization process at the cluster level, including how the blocks are assigned to intervention and control arms.

 We have discussed the process of randomization and block assignment.

 Page no.: 8

 Line no.: 186-193

 Mention how many clusters will be in each arm after randomization.

 Mentioned.

 Page no.: 8

 Line no.: 186-193

 Clarify whether the pilot test and feedback phase will include both intervention and control groups or only the intervention group.

 We have clarified regarding the pilot testing.

 Page no.: 7

 Line no.: 161-163

 Specify how missing data will be handled in the analysis.

 We elaborated on the handling of missing data.

 Page no.: 11

 Line no.: 252-255

 Provide a brief explanation of hierarchical regression and its role in the analysis.

 We have explained the role of hierarchical regression in the analysis.

 Page no.: 11

 Line no.: 267-271

 Kindly state the potential strenghts and limitations you may encounter while carrying out this study and how you intend to circumvent these.

 We have added strengths, limitations, and mitigation strategies

 Page no.: 12

 Line no.: 283-294

**References**

 I strongly encourage the use of more recent literature and citations.

 Majority of the literature cited in this manuscript were published within past 15 years.
